# Plasma miRNA Biomarker Signatures in Parkinsonian Syndromes

**DOI:** 10.1007/s12035-025-04890-w

**Published:** 2025-04-04

**Authors:** Stylianos Ravanidis, Anastasia Bougea, Christos Koros, Athina-Maria Simitsi, Panagiotis Kokotis, Leonidas Stefanis, Epaminondas Doxakis

**Affiliations:** 1 Center of Basic Research, Biomedical Research Foundation of the Academy of Athens, 11527 Athens, Greece; 2 Center of Clinical Research, Biomedical Research Foundation of the Academy of Athens, 11527 Athens, Greece; 3 First Department of Neurology, National and Kapodistrian University of Athens Medical School, 11528 Athens, Greece

**Keywords:** Parkinson’s disease (PD), Progressive supranuclear palsy (PSP), Multiple system atrophy (MSA), MicroRNA (miRNA), Plasma, Biomarkers, Diagnosis

## Abstract

Diagnosing atypical parkinsonian syndromes (APS) remains challenging due to overlapping clinical features and limited diagnostic tools. Brain-enriched microRNAs (miRNAs), which regulate neuronal development and function, are detectable in plasma and could serve as molecular biomarkers. This prospective study aimed to identify plasma brain-enriched miRNAs that can distinguish APS and elucidate affected molecular pathways. Reverse transcription-quantitative PCR (RT-qPCR) was performed on plasma samples from patients with idiopathic Parkinson’s disease (iPD), multiple system atrophy (MSA), including the cerebellar subtype (MSA-C) and the parkinsonian subtype (MSA-P), progressive supranuclear palsy (PSP), and healthy controls. MiRNA expression analysis revealed distinct molecular fingerprints for each parkinsonian syndrome, with opposite trends between MSA and iPD compared to controls, suggesting distinct pathogenic mechanisms. Most dysregulated miRNAs clustered at chromosome (Chr)14q32 and shared binding sites for CREB1, CEBPB, and MAZ transcription factors. Pathway analysis revealed enrichment in prion diseases, Hippo signaling, TGF-beta signaling, and FoxO signaling pathways.

**Supplementary Information:**

The online version contains supplementary material available at 10.1007/s12035-025-04890-w.

## Introduction

Parkinsonian syndromes comprise a spectrum of progressive neurodegenerative movement disorders characterized by a core triad of symptoms: rigidity, tremors, and bradykinesia. The clinical picture extends beyond motor manifestations, encompassing diverse non-motor symptoms such as cognitive decline, sleep disturbances, and autonomic dysfunction. Pathologically, parkinsonian syndromes are characterized by abnormal accumulation of protein aggregates within brain cells, particularly α-synuclein and tau, leading to neurodegeneration and cellular dysfunction.

Parkinsonian syndromes encompass Parkinson’s disease (PD), dementia with Lewy bodies (DLB), Parkinson’s disease dementia (PDD), and multiple system atrophy (MSA) as synucleinopathies, as well as progressive supranuclear palsy (PSP) and corticobasal syndrome (CBS) as tauopathies. PD, with an estimated incidence of 1–2 per 1000 individuals, results from the progressive loss of dopaminergic neurons in the substantia nigra pars compacta [1]. It has familial and sporadic forms, with abnormal accumulation of misfolded α-synuclein in neurons as a cellular hallmark. The estimated prevalence of PSP and MSA is 6.4 and 4.4 per 100,000 population, respectively [2]. PSP, a tauopathy affecting the brainstem and basal ganglia, causes postural instability, vertical gaze palsy, dysarthria, dysphagia, and cognitive decline. MSA is characterized by α-synuclein accumulation in oligodendrocytes, affecting multiple systems, particularly autonomic function, leading to orthostatic hypotension and genitourinary dysfunction. MSA is classified into two subtypes based on symptoms: MSA-C (cerebellar type), characterized by ponto-cerebellar degeneration and ataxia, and MSA-P (parkinsonian type), which primarily affects the basal ganglia and presents with PD-like symptoms. Unlike PD, PSP and MSA follow a relentless progression unresponsive to levodopa, with a median survival of 5–10 years [3, 4].

The accurate differentiation of PD from atypical parkinsonian syndromes (APS) remains a major clinical challenge due to overlapping symptoms and the absence of definitive diagnostic tools. Misdiagnosis rates can be as high as 24% in APS cases, as revealed by postmortem studies, underscoring the need for reliable biomarkers [5]. While clinical assessments and neuroimaging techniques—such as single-photon emission computed tomography (SPECT), positron emission tomography (PET), and advanced magnetic resonance imaging (MRI)—aid in diagnosis, their accuracy in distinguishing PD from APS remains suboptimal [6, 7]. Automated 18F-fluorodeoxyglucose PET (FDG-PET) classification has improved diagnostic performance, yet it is not widely implemented [8]. Beyond neuroimaging, several molecular and functional biomarkers have been explored, including seed amplification assays (SAAs), which demonstrate high specificity for synucleinopathies but face standardization challenges and slightly lower sensitivity for MSA [9, 10]. Autonomic testing has also been assessed; however, it has limited accuracy in differentiating APS subtypes, as it poorly distinguishes PSP from controls and PD from MSA patients [11–13]. Additionally, phosphorylated α-synuclein detection in skin biopsies has emerged as a promising diagnostic marker for detecting synucleinopathies and may help in differentiating PD from MSA but is not yet widely adopted in routine clinical practice [14, 15]. Another potential biomarker source is CNS-enriched extracellular vesicles (EVs), which contain disease-relevant proteins and non-coding RNAs. Despite promising initial findings, EV-based biomarkers suffer from challenges related to reproducibility and publication bias [16, 17]. Given these limitations, minimally invasive biomarkers such as circulating miRNAs may offer a valuable alternative for accurate diagnosis.

MiRNAs, ~ 22 nucleotide-long non-coding RNA molecules, regulate gene expression by binding to complementary sequences within target mRNAs, leading to translational repression or degradation [18]. Although intracellular, miRNAs are secreted in complex with Argonaute proteins or within exosomes for intercellular communication and are detected in biofluids, including plasma, serum, and cerebrospinal fluid, enabling their analysis as minimally invasive biomarkers. Each miRNA simultaneously regulates multiple mRNAs, orchestrating complex gene regulatory networks that influence development and pathological processes. The human genome encodes 2654 mature miRNA sequences predicted to regulate over 60% of protein-coding genes [19]. Various miRNAs exhibit tissue specificity, which is crucial in maintaining cellular homeostasis and contributing to organ-specific functions. In PD, several miRNAs have been shown to target pathogenic proteins [20, 21] or modulate relevant pathways [22], be dysregulated upon pathogenic protein expression, or exhibit altered expression in patient brain tissue [23] or biofluids (reviewed in [24]). As key gene regulators in the brain, altered miRNA levels will likely contribute to the motor and non-motor symptoms of PD and related disorders.

While most miRNA biomarker studies have focused on PD, few describe expression differences between PD and atypical parkinsonisms, like MSA or PSP [25–31]. This study profiled miRNAs in plasma samples from PD, MSA, and PSP patients to establish diagnostic signatures and elucidate underlying regulatory networks.

## Subjects and Methods

### Study Population

The study included 25 iPD patients, 13 MSA-C patients, 12 MSA-P patients, 11 PSP patients, and 25 healthy individuals (HC). PSP patients met the Movement Disorder Society (MDS) clinical diagnostic criteria for PSP (MDS-PSP criteria), specifically the Richardson’s syndrome (PSP-RS) phenotype [32]. MSA patients were diagnosed according to established criteria [33]. PSP and MSA were sporadic in all cases. Patients underwent brain MRI or computed tomography (CT), and no relevant brain vascular lesions that could explain the clinical phenotype were detected. Medical records provided patient background information. The control group comprised spouses or unrelated companions without known neurological disease, comorbidities, or family history of PD. Individuals with concurrent malignant tumors, psychiatric disorders, collagen diseases, endocrine and cardiovascular diseases, or infections were excluded from both patient and control groups, as these conditions are expected to affect the expression profile of circulating miRNAs. Additionally, individuals suffering from other neurological conditions, including dementia and Parkinsonism, were excluded from the control group. All patients and controls were recruited from the National and Kapodistrian University of Athens’ First Department of Neurology at Eginition Hospital (protocol approval #198, ADA: ΨΒ0746ΨΟΝ2-ΡΘ2). Two neurologists diagnosed PD according to the criteria established by Postuma et al. [34]. Essential demographic and clinical information, including motor and non-motor manifestations of the disease, and rating scales were collected and documented (see below).

### Assessment Scales

This study employed a comprehensive battery of assessments to evaluate patients’ cognitive, motor, and autonomic functions. The Unified Multiple System Atrophy Rating Scale (UMSARS), Progressive Supranuclear Palsy Rating Scale (PSPRS), Frontal Assessment Battery (FAB), Digit Span Test (forward and backward versions), Schwab and England Activities of Daily Living Scale (SE), Hoehn and Yahr staging (H&Y), and Goldenberg apraxia scale (GB total, including subcategories like imitation of hands, fingers, pantomime, Grip, and position-movement) assessed various aspects of motor and cognitive function. Additionally, the study included the Montreal Cognitive Assessment (MoCA), animal fluency test, Geriatric Depression Scale (GDS), Movement Disorder Society-Unified PD Rating Scale (MDS-UPDRS III), and Scales for Outcomes in PD-Autonomic Questionnaire (SCOPA-AUT, including subscales for various autonomic functions). Levodopa equivalent daily dose (LEDD) was also recorded. Furthermore, detailed assessment of autonomic function was conducted through tests examining cardiovagal innervation (heart rate (HR) response to deep breathing, Valsalva ratio, and blood pressure (BP) responses to standing (systolic blood pressure (subSBP), diastolic blood pressure (subDBP), and 30:15 ratio) and adrenergic innervation (beat-to-beat BP responses to Valsalva maneuver, DBP difference in hand grip, and DBP change in HR).

### Plasma Collection

Venous blood samples were collected in EDTA-treated tubes (367525, BD Vacutainer) and processed immediately. Samples were centrifuged at 1500 × g for 5 min at 4 °C, and the supernatant plasma was aliquoted into 1 mL fractions and stored at − 80 °C until analysis.

### MiRNA Isolation from Plasma and Reverse Transcription Quantitative PCR (RT-qPCR) Analysis

Plasma samples were thawed at room temperature and centrifuged at 4 °C to pellet any cell debris. Samples were analyzed spectrophotometrically for oxyhemoglobin absorbance at 414 nm, with a cut-off level set at 0.22 against water. Approximately 15% of plasma samples were discarded due to hemolysis. For miRNA extraction, 300 μL of plasma was processed using the NucleoSpin® miRNA plasma kit (740,981, Macherey–Nagel) in duplicate according to the manufacturer’s instructions. To improve miRNA yield, 1 μg of MS2 RNA (10,165,948,001, Roche) was added to each sample before extraction. Polyadenylation and RT-PCR reactions were performed in triplicate. qPCR was conducted using SYBR FAST Universal 2X qPCR Master Mix (KK4618, Kapa Biosystems). The analysis included established reference miRNAs (miR-191-5p, miR-425-5p, miR-423-3p) validated for their stability in plasma across multiple independent studies [35, 36]. These were confirmed as the most stable from our initial panel of five reference miRNAs (miR-103a-3p, miR-191-5p, miR-425-5p, miR-223-3p, miR-423-3p) used in our previous work [37, 38]. Sample quality was assessed using the miR-23a/miR-451a hemolysis indicator pair, with samples exceeding a Δ(Ct23α−3p-Ct451α) of 5.5 excluded from the analysis [39, 40]. Primer sequences are provided in Supplemental Table 1. The relative expression level of miRNAs was calculated using the 2-ΔΔCt method.

### List of miRNAs

The miRNA panel was established through a systematic multi-step selection process. Twenty-four brain-enriched miRNAs were selected based on the following criteria: (1) consistently high brain expression demonstrated in at least three independent genome-wide tissue-specific surveys [41–44], (2) our in vitro validation through human tissue-wide expression analysis [38], and (3) reliable detectability in plasma (Ct values < 30) [38]. These were miR-7-5p, miR-124-3p, miR-127-3p, miR-128-3p, miR-132-3p, miR-136-3p, miR-153-3p, miR-154-5p, miR-219a-5p, miR-323a-3p, miR-329-5p, miR-330-3p, miR-338-3p, miR-382-5p, miR-409-3p, miR-410-3p, miR-411-5p, miR-432-5p, miR-487b-3p, miR-495-3p, miR-598-3p, miR-654-3p, miR-885-5p, and miR-3200-3p. Additionally, miR-19b-3p, miR-29a-3p, and miR-106a-5p were included due to their previously reported dysregulation in MSA or PSP [25, 27, 28, 30, 31]. Finally, miR-22-3p was added because it targets glucosylceramidase beta 1 (*GBA1*) mRNA and exhibits high brain expression [21].

### Chromosomal Location of miRNAs

Genomic coordinates for each human miRNA were obtained from the miRbase release 22.1 website [19]. The coordinates were uploaded to the PhenoGram software to visualize data, which was used to create the chromosomal ideogram [45].

### Transcription Factors Regulating miRNAs

The TransmiR v2.0 literature-curated database of experimentally validated transcription factors (TFs)-miRNA regulations was searched to identify the TFs located 300 bp upstream and 100 bp downstream of each dysregulated miRNA Transcription start site (TSS) [46].

### Pathway Analysis

The DIANA mirPath v.3 software suite was used to identify miRNA-regulated pathways. This software makes the functional annotation of one or more miRNAs possible using standard hypergeometric distributions, unbiased empirical distributions, and meta-analysis statistics [47]. Here, predicted targets from the DIANA microT-CDS algorithm with high-quality experimentally supported interactions were used to identify the Kyoto encyclopedia of genes and genomes (KEGG) molecular pathways targeted by each miRNA. The combinatorial effect of dysregulated miRNAs was identified by simultaneously selecting multiple miRNAs in the software. The default values (FDR corrected *p*-value threshold 0.05, microT-CDS threshold 0.8) were used for the analysis.

### Statistical Analysis

Statistical analysis was performed using GraphPad PRISM v5.0, R v3.5.3, and MedCalc 23.1.7. The Shapiro–Wilk test assessed the normality of miRNA distributions. Most miRNAs were non-normally distributed; therefore, non-parametric tests were employed. The Kruskal–Wallis test was used to compare miRNA distributions across patient groups (MSA-C, MSA-P, PSP, iPD, HC). Post-hoc pairwise comparisons were performed using Dunn’s test with Benjamini–Hochberg correction for multiple comparisons. Spearman’s correlation with Bonferroni correction assessed correlations between miRNA expression and participants’ characteristics. Receiver operating characteristic (ROC) curves and area under the curve (AUC) were calculated to evaluate the predictive accuracy of plasma miRNAs for differentiating parkinsonian syndromes. For group comparisons, a stepwise logistic regression model was employed (*p*-value for variable entry = 0.15, *p-*value for removal = 0.20). Standard error was calculated using the DeLong et al. approach [48]. MiRNAs with significant differential expression were selected as candidate classifiers for ROC analysis. When no significant differences were found, a less stringent *p*-value (*p* < 0.2) was used while adjusting for sex and age.

### Data Availability

Data supporting the results are available from the corresponding author upon reasonable request and are subject to data privacy regulations and ethical considerations.

## Results

### Study Population and Rating Scales

The demographic characteristics of 25 healthy controls, 25 iPD patients, 13 MSA-C patients, 12 MSA-P patients, and 11 PSP patients are summarized in Table 1. No significant differences were observed in age, sex ratio, education, disease duration, age at onset, or levodopa equivalent daily dose (LEDD) among the groups. No significant differences were found among parkinsonian syndromes in several scales, including clinical UPDRS III, UMSARS (total, parts 1, 2, and 4), and FAB scales. However, differences were observed in SCOPA-AUT (gastrointestinal, urinary, cardiovascular, and sexual function in men), SE, and HY. MSA patients exhibited more prominent autonomic nervous system symptoms, particularly in the gastrointestinal, urinary, and cardiovascular systems, compared to iPD and PSP patients. Regarding neuropsychological scales, significant differences among subgroups were limited to the MoCA, animal fluency test, GB Position/Movement task, and GDS. PSP patients demonstrated significantly lower scores on the MoCA, animal fluency test, and GB Position/Movement task (Table 2).

**Table 1:** Demographic characteristics of healthy controls and patients with iPD, MSA-C, MSA-P, or PSP

	MSA-C	MSA-P	PSP	iPD	Control	*p*-value
No of subjects	13	12	11	25	25	
Age [mean (SD)]	62.77 (8.37)	62.08 (7.56)	66.64 (8.20)	62.36 (7.85)	62.48 (7.39)	0.5871
Sex (male/female)	7/6	4/8	6/5	10/15	11/14	0.7872
Education [mean (SD)]	12.23 (4.07)	11.42 (4.85)	12.64 (4.76)	12.40 (3.96)	13.36 (4.73)	0.7878
Age at onset [mean (SD)]	58.3 (9.04)	57.8 (7.8)	61.09 (7.7)	56.05 (10.04)	-	0.5523
Disease duration [median (IQR)]	5 (4)	3.5 (4)	4 (8)	4 (6)	-	0.9341

Normally distributed variables are presented as mean (SD), while non-normally distributed variables are presented as median (IQR). For normally distributed variables, the null hypothesis states that the means are equal across groups, tested using one-way analysis of variance (ANOVA). Rejection of this hypothesis indicates significant differences in means among groups. For non-normally distributed variables, the null hypothesis posits that the variable’s distribution is identical across groups, tested using the Kruskal–Wallis test. Rejection of this hypothesis indicates significant differences in the variable’s distribution among groups. The significance level for these tests is set at *α* = 0.05

**Table 2:** Comparison of clinical and neuropsychological measures among patients with iPD, MSA-C, MSA-P, or PSP

Measure	Variable	MSA-C	MSA-P	PSP	iPD	*p*-value	Effect size
UPDRS III	Mean (SD)	32.08 (20.02)	40.83 (22.18)	38.0 (18.93)	24.64 (19.24)	0.0925	0.106
LEDD	Mean (SD)	535.54 (533.16)	625.17 (398.36)	647.18 (377.75)	761.20 (657.82)	0.6647	0.027
PSP score	Mean (SD)	25.92 (12.28)	32.83 (16.89)	34.09 (12.58)	-	0.3120	0.068
UMSARS total	Mean (SD)	43.92 (21.68)	45.75 (22.25)	45.0 (20.91)	-	0.9777	0.001
UMSARS 1	Mean (SD)	23.15 (11.34)	24.25 (11.48)	20.36 (12.50)	-	0.7203	0.020
UMSARS 2	Mean (SD)	18.15 (10.21)	18.75 (10.98)	21.91 (8.57)	-	0.6298	0.028
UMSARS 4	Median (IQR)	2.0 (1.0)	3.0 (1.0)	3.0 (1.0)	-	0.6918	− 0.036
SCOPA total	Median (IQR)	20.0 (11.0)	19.5 (15.0)	5.5 (9.0)	2.0 (2.0)	**0.0001**^**b,d**^	0.659
SCOPA gastro	Median (IQR)	5.0 (8.0)	9.5 (6.0)	3.5 (6.0)	0.5 (2.0)	**0.0001**^**b,d,f**^	0.380
SCOPA urinary	Median (IQR)	8.0 (10.0)	5.5 (11.0)	1.5 (4.0)	0.0 (1.5)	**0.0001**^**b,d**^	0.386
SCOPA cardio	Median (IQR)	1.0 (4.0)	0.0 (1.5)	0.0 (0.0)	0.0 (0.0)	**0.0019**^**b,c**^	0.205
SCOPA thermo	Median (IQR)	0.0 (1.0)	0.0 (2.0)	0.0 (0.0)	0.0 (0.0)	0.0971	0.057
SCOPA pupillo	Median (IQR)	0.0 (0.0)	0.0 (0.0)	0.0 (0.0)	0.0 (0.0)	0.1336	0.045
SCOPA sex men	Median (IQR)	4.0 (6.0)	6.0 (2.0)	0.0 (0.0)	0.0 (0.0)	**0.0007**^**b,d,e**^	0.472
SE	Median (IQR)	70.0 (20.0)	60.0 (20.0)	60.0 (20.0)	80.0 (20.0)	**0.0006**^**d,f**^	0.241
H&Y	Median (IQR)	3.0 (1.0)	3.0 (1.0)	3.0 (1.0)	1.0 (1.0)	**0.0002**^**b,d,f**^	0.284
MOCA	Median (IQR)	20.0 (7.0)	23.5 (5.5)	13.0 (12.0)	24.0 (8.0)	0.0100	0.139
FAB	Median (IQR)	13.0 (4.0)	14.5 (5.0)	12.0 (6.0)	12.0 (4.0)	0.6706	− 0.024
Animal fluency	Mean (SD)	5.77 (2.09)	6.08 (2.02)	4.18 (2.86)	9.04 (4.32)	**0.0006**^**b,f**^	0.260
Digit Span total	Median (IQR)	14.0 (8.0)	15.0 (12.0)	8.0 (14.0)	-	0.1403	0.055
Digit Span backwards	Median (IQR)	6.0 (4.0)	7.0 (7.0)	4.0 (7.0)	-	0.1682	0.045
Digit Span forwards	Median (IQR)	8.0 (4.0)	8.0 (7.0)	4.0 (7.0)	-	0.1350	0.057
GB total score	Mean (SD)	106.23 (23.16)	96.75 (20.72)	83.73 (27.17)	-	0.0828	0.140
GB imitation score	Median (IQR)	73.0 (17.0)	65.0 (26.0)	54.0 (41.0)	-	0.1894	0.038
GB hand imitation	Median (IQR)	38.0 (8.0)	36.0 (13.5)	28.0 (20.0)	-	0.1902	0.038
GB finger imitation	Mean (SD)	33.08 (8.04)	31.17 (7.54)	25.91 (10.37)	-	0.1336	0.115
GB pantomime score	Mean (SD)	38.23 (11.58)	31.42 (7.74)	30.18 (8.54)	-	0.0931	0.134
GB grip	Mean (SD)	15.08 (4.41)	13.83 (3.41)	14.09 (2.88)	-	0.6722	0.024
GB position/movement	Mean (SD)	23.15 (7.80)	17.58 (5.62)	16.18 (6.90)	-	**0.0399**^**c**^	0.177
GDS	Mean (SD)	8.00 (2.80)	8.25 (4.58)	8.27 (3.23)	4.56 (2.95)	**0.0017**^**b,d,f**^	0.232
subSBP*	Mean (SD)	26.60 (16.32)	22.04 (20.73)	-	-	0.632	0.101
subDBP*	Mean (SD)	11.60 (7.52)	8.73 (8.32)	-	-	0.481	0.139
Ratio 30:15*	Mean (SD)	1.05 (0.08)	1.13 (0.13)	-	-	0.176	−0.345
DBP change in HR*	Mean (SD)	6.67 (1.63)	7.27 (3.40)	-	-	0.698	−0.143
DBP difference in hand grip *	Mean (SD)	10.67 (5.28)	10.47 (5.16)	-	-	0.944	0.015
Valsalva ratio*	Mean (SD)	1.27 (0.12)	1.38 (0.23)	-	-	0.316	−0.372

Normally distributed variables are presented as mean (SD), while non-normally distributed variables are presented as median (IQR). For normally distributed variables, the null hypothesis states that the means are equal across groups, tested using one-way analysis of variance (ANOVA). Rejection of this hypothesis indicates significant differences in means among groups. For non-normally distributed variables, the null hypothesis posits that the variable’s distribution is identical across groups, tested using the Kruskal–Wallis test. Rejection of this hypothesis indicates significant differences in the variable’s distribution among groups. The significance level for these tests is set at *α* = 0.05. Post-hoc pairwise comparisons were conducted using Tukey’s test for normally distributed variables and Dunn’s test for non-normally distributed variables. Statistically significant group differences are indicated by superscript letters corresponding to specific comparisons

^a^MSA-C vs MSA-P

^b^MSA-C vs iPD

^c^MSA-C vs PSP

^d^MSA-P vs iPD

^e^MSA-P vs PSP

^f^iPD vs PSP

Effect sizes were calculated as follows: *η*^2^ for ANOVA, ε^2^ for Kruskal–Wallis tests, and Cohen’s *d* for independent samples Student t-test

^*^Independent samples Student t-test

### Distinct Plasma miRNA Profiles in Parkinsonian Syndromes

**Fig. 1:**
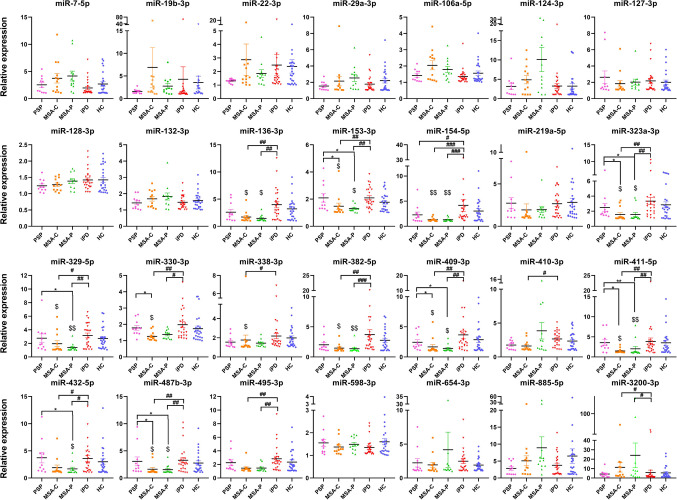
Plasma miRNA expression levels in healthy controls and patients with iPD, MSA-C, MSA-P, or PSP. Each graph depicts the mean ± SEM for the relative expression of specific miRNAs. Pairwise comparisons between groups were performed using Dunn’s test with Benjamini–Hochberg correction for multiple comparisons. Asterisks (*) indicate significant differences compared to PSP: *p < 0.05, **p < 0.01, ***p < 0.001; hash marks (#) indicate significant differences compared to iPD: #*p* < 0.05, ##*p* < 0.01; dollar signs ($) indicate significant differences compared to HC: $*p* < 0.05, $$*p* < 0.01

RT-qPCR analysis revealed distinctive miRNA expression patterns across different parkinsonian syndromes. The analysis of plasma samples from healthy controls and patient cohorts examined 24 brain-enriched miRNAs along with 4 ubiquitous miRNAs (miR-19b-3p, miR-29a-3p, miR-106a-5p, and miR-22-3p) (Fig. 1, Supplementary Table 2).

The most striking findings emerged from the MSA cohorts, where both variants (MSA-C and MSA-P) exhibited significant downregulation of multiple miRNAs compared to other groups. Most notably, miR-153-3p, miR-323a-3p, miR-409-3p, miR-411-5p, and miR-487b-3p were consistently reduced in MSA compared to controls, iPD, and PSP patients. The remarkable similarity in expression patterns between MSA-C and MSA-P suggests shared underlying pathological mechanisms.

In contrast, iPD samples displayed distinctly higher expression of several miRNAs compared to MSA variants, particularly miR-154-5p, miR-382-5p, and miR-495-3p. These opposing patterns likely reflect fundamental differences in disease processes between iPD and MSA. PSP samples demonstrated intermediate expression levels for many miRNAs, with key differences observed in miR-154-5p (reduced compared to iPD) and several MSA-associated miRNAs.

The findings reveal widespread downregulation of brain-enriched miRNAs specifically in MSA, distinguishing it from other parkinsonian syndromes. Despite their clinical similarities, the consistent differences between MSA and iPD profiles indicate distinct underlying pathological mechanisms.

### Association Between miRNA Levels and Clinical Features

**Fig. 2:**
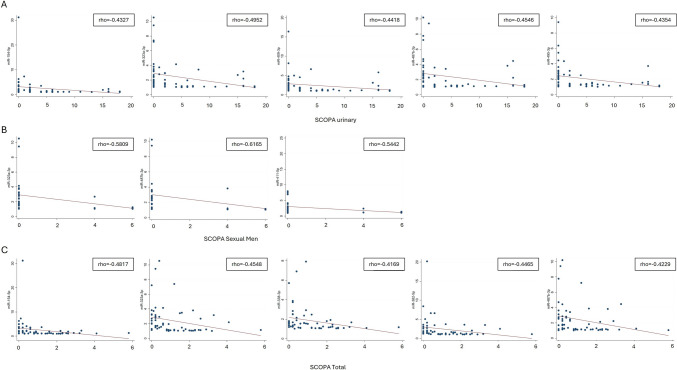
Correlation of selected miRNAs with SCOPA scales. MiR-154-5p, miR-323a-3p, miR-338-3p, miR-382-5p, miR-409-3p, miR-411-5p, miR-487b-3p, and miR-495-3p show significant correlations with SCOPA scores (**A**, SCOPA urinary; **B**, SCOPA sexual men; **C**, SCOPA total). Values presented are Spearman’s rank correlation coefficients (*r*) and corresponding *p-*values. After Bonferroni correction for multiple comparisons, the significance threshold was set at *p* < 0.0017

Spearman correlation analysis related miRNA levels to clinicodemographic features. No correlations were found with age, disease onset/duration, or UPDRS III score (Supplementary Table 3). The only significant correlation occurred between miR-29a-3p and LEDD in the MSA-C subgroup (rho = 0.78, *p* = 0.0017, Supplementary Fig. 1). Correlations between miRNA expression and cognitive/motor assessment scores were analyzed, including MoCA, FAB, Digit Span Total, Goldenberg Scale, PSP score, UMSARS Total, SCOPA Total, H&Y stage, and autonomic function measures (Supplementary Table 4). Significant negative correlations emerged between SCOPA-AUT and levels of several miRNAs: miR-154-5p (SCOPA urinary and total), miR-323a-3p (urinary, sexual men, and total), miR-338-3p (total), miR-382-5p (total), miR-409-3p (urinary), miR-411-5p (sexual men), miR-487b-3p (urinary, sexual men, and total), and miR-495-3p (urinary). These findings suggest that the identified miRNAs may contribute to maintaining autonomic nervous system homeostasis and potentially underlie the autonomic dysfunction observed in parkinsonian syndromes (Fig. 2).

### Discriminant Analysis Based on Plasma miRNA Levels

**Fig. 3:**
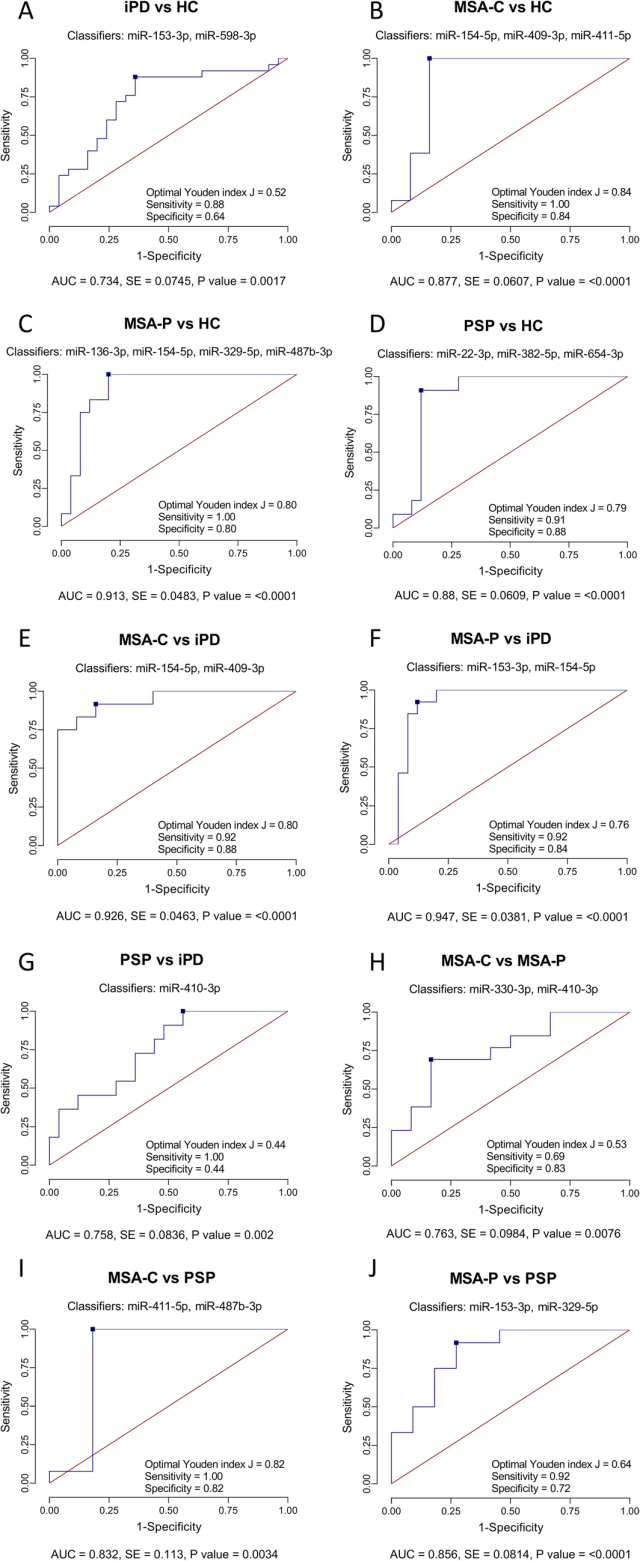
Receiver operating characteristic (ROC) curve analysis for discriminating between patient groups and healthy controls using plasma miRNA expression profiles.** A** iPD vs HC, **B** MSA-C vs HC, **C** MSA-P vs HC, **D** PSP vs HC, **E** MSA-C vs iPD, **F** MSA-P vs iPD, **G** PSP vs iPD, **H** MSA-C vs MSA-P, **I** MSA-C vs PSP, and **J** MSA-P vs PSP. Sensitivity and specificity are reported at the optimal Youden’s index cutoff—denoted by a blue square on the curve. *P* values are calculated against an AUC of 0.5

ROC curve analysis was performed to evaluate the utility of plasma miRNA levels for discriminating between parkinsonian syndromes and healthy controls and among the syndromes themselves. For distinguishing iPD patients from healthy controls, a two-miRNA panel (miR-153-3p, miR-598-3p) yielded an AUC of 0.73 (Fig. 3A). For identifying MSA-C, a three-miRNA panel (miR-154-5p, miR-409-3p, and miR-411-5p) achieved an AUC of 0.88, while a four-miRNA panel (miR-136-3p, miR-154-5p, miR-329-5p, and miR-487b-3p) yielded an AUC of 0.91 for MSA-P detection (Fig. 3B, C). For PSP, miR-22-3p, miR-382-5p, and miR-654-3p provided an AUC of 0.88 compared to controls (Fig. 3D).

For differential diagnosis among parkinsonian syndromes, miR-154-5p and miR-409-3p distinguished iPD from MSA-C with an AUC of 0.93, and miR-153-3p and miR-154-5p discriminated iPD from MSA-P with an AUC of 0.95 (Fig. 3E, F). MiR-410-3p alone distinguished between PSP from iPD with an AUC of 0.76, while miR-330-3p and miR-410-3p differentiated MSA-C from MSA-P with an AUC of 0.76 (Fig. 3G, H). miR-411-5p and miR-487b-3p distinguished MSA-C from PSP with an AUC of 0.83, while miR-153-3p and miR-329-5p discriminated MSA-P from PSP with an AUC of 0.86 (Fig. 3I, J).

### Chromosomal Distribution and Transcription Factors

**Fig. 4:**
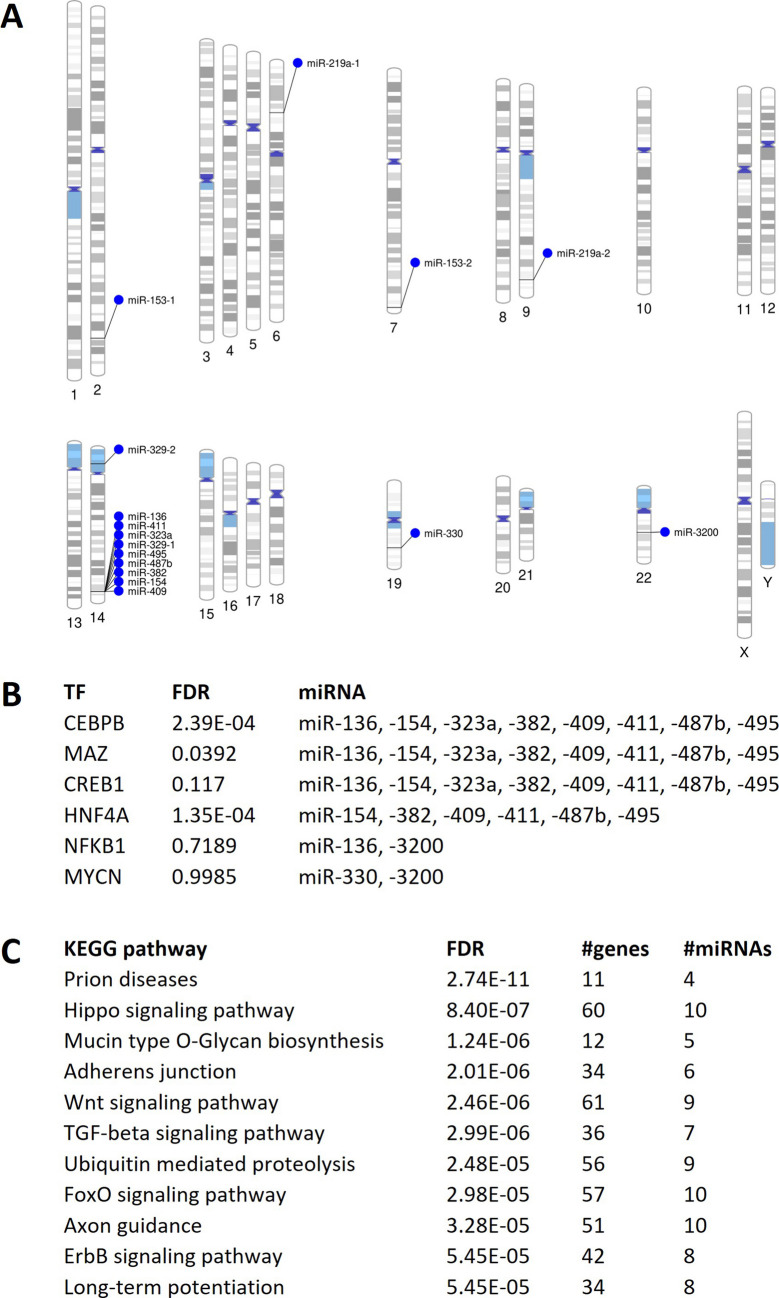
Genomic context and functional implications of dysregulated miRNAs in Parkinsonism.** A** Phenogram illustrating the chromosomal locations of dysregulated miRNAs. **B** Transcription factors regulating the expression of two or more dysregulated miRNAs. **C** KEGG pathway analysis of the union of dysregulated miRNA targets. The number of genes each miRNA regulates within a given pathway is indicated in parentheses

Genomic loci of differentially expressed miRNAs were identified to understand their regulatory mechanisms. Chromosomal coordinates of dysregulated miRNAs were retrieved from miRbase 22.1 and visualized using Phenogram (Fig. 4A). Notably, over half of these miRNAs (miR-136, miR-154, miR-323a, miR-329–1, miR-382, miR-409, miR-411, miR-487b, and miR-495) clustered at the same locus on chromosome (Chr)14q32. This finding suggests potential co-regulation by specific transcription factors or methylation events in this region.

To identify candidate transcription factors regulating these differentially expressed miRNAs, the manually curated TransmiR v2.0 database was employed with stringent parameters, searching for transcription factor binding sites (TFBS) within the promoter regions of each miRNA. This analysis revealed an enrichment of TFBS for CCAAT enhancer binding protein beta (CEBPB), MYC-associated zinc finger protein (MAZ), and cAMP-responsive element binding protein 1 (CREB1) within half of the analyzed miRNAs, notably including eight out of the nine miRNAs located on chromosome 14 (Fig. 4B). The clustering of over half of the differentially expressed miRNAs at Chr14q32 and common transcription factor binding sites in these miRNAs suggest coordinated regulation that may contribute to the aberrant miRNA expression profiles observed in MSA.

### Pathway Analysis

DIANA mirPath v3 tool was used to identify impacted biological pathways. Dysregulated miRNA targets showed significant enrichment in 49 KEGG categories. Several pathways stood out with highly significant *p*-values (Fig. 4C). These were “Prion diseases” (*p* < 2.7 × 10^−11^, 11 genes), “Hippo signaling pathway” (*p* < 8.4 × 10^–7^, 60 genes), “Mucin type O-Glycan biosynthesis” (*p* < 1.2 × 10^−6^, 12 genes), “Adherens junction” (*p* < 2.0 × 10^−6^, 34 genes), “Wnt signaling pathway” (*p* < 2.5 × 10^−6^, 20 genes), “TGF-beta signaling pathway” (*p* < 3.0 × 10^−6^, 36 genes), “Ubiquitin mediated proteolysis” (*p* < 2.5 × 10^−5^, 56 genes), and “FoxO signaling pathway” (*p* < 3.0 × 10^−5^, 57 genes) (Fig. 4C and Supplementary Table 5 for the complete list).

## Discussion

Brain-enriched miRNAs have emerged as promising biomarkers in parkinsonian syndromes. Previous work established their utility in distinguishing healthy controls from genetic and idiopathic PD subjects [37, 38], with comprehensive reviews highlighting their potential in PD biomarker research [24]. The present study expanded this approach to MSA and PSP, two rarer parkinsonian syndromes that present significant diagnostic challenges. Importantly, the findings in iPD versus healthy controls replicated previous studies [37, 38], with 14 out of 16 differentially expressed miRNAs showing consistent patterns despite using a refined set of reference miRNAs. This reproducibility, combined with the observed consistent expression patterns between MSA-C and MSA-P subgroups across multiple miRNAs (notably in miR-136-3p, miR-154-5p, miR-323a-3p, and miR-487b-3p), strengthens confidence in the results despite the relatively small sample sizes.

MSA and PSP patients differed significantly from iPD patients in several clinical and neuropsychological scales, including SCOPA, SE, and HY. Both MSA-C and MSA-P cohorts showed pronounced involvement of the gastrointestinal and urinary systems, with additional cardiovascular issues in MSA-C patients. In neuropsychological assessments, PSP patients scored lower on the MoCA and animal fluency tests, while both MSA subtypes displayed similar performance, scoring higher than PSP but lower than iPD patients. As measured by GDS, dystonia severity was comparable across all atypical parkinsonisms. However, PSP patients scored lowest on the GBS position/movement subscale, followed closely by MSA-P patients. Finally, PSP patients exhibited more pronounced depressive symptomatology.

The plasma miRNA expression analysis revealed distinct molecular signatures, with notably opposite trends between MSA and iPD compared to controls. Six brain-enriched miRNAs differentiated MSA from controls, and eleven distinguished MSA from iPD. Their alignment with previous studies supports the robustness of these findings despite methodological differences. For instance, Ramaswamy et al. reported identical expression patterns for several miRNAs in PSP, including miR-19b-3p, miR-106a-5p, miR-136-3p, and miR-154-5p, although they used different reference controls (miR-16) [29]. In MSA studies, Kume et al. demonstrated similar upregulation of miR-106a-3p in serum samples [25], while Uwatoko et al.’s plasma analysis showed comparable expression patterns for miR-19b-3p in both MSA subtypes and iPD [31]. Additionally, the findings align with Perez-Soriano et al.’s CSF study showing upregulation of miR-106a-3p in MSA patients [28]. Notably, while fewer miRNAs reached significance for PSP and iPD compared to controls—possibly reflecting sample size constraints and disease severity differences—robust classification was still observed in specific comparisons. For instance, miR-153-3p and miR-598-3p separated iPD from healthy controls (AUC = 0.734), whereas miR-22-3p, miR-382-5p, and miR-654-3p distinguished PSP from healthy controls (AUC = 0.88). These findings, alongside the reproducibility of iPD profiles compared to previous studies and the consistent expression patterns between MSA-P and MSA-C, reinforce the biological relevance of these results.

Despite advances in biomarker discovery, current diagnostic tools for parkinsonian syndromes remain imperfect. Neuroimaging, SAAs, and phosphorylated α-synuclein in skin biopsies provide valuable insights but face limitations in accuracy, standardization, or feasibility for routine use. In this context, circulating miRNAs offer a complementary, minimally invasive approach with unique advantages. MiRNAs are stable in biofluids, protected from degradation within exosomes or Argonaute complexes [49], and quantifiable by rapid RT-qPCR methods available in most clinical laboratories. Unlike protein biomarkers, mature miRNA levels reflect actual regulatory activity, providing mechanistic insights potentially relevant to clinical trials.

Several miRNA panels demonstrated strong diagnostic performance in this study across multiple comparisons. MSA-P and MSA-C were differentiated from controls with AUCs of 0.913 and 0.877, respectively, and iPD with 0.947 (MSA-P) and 0.926 (MSA-C). Moreover, MSA-C and MSA-P were separated from PSP (AUC = 0.832 and AUC = 0.856, respectively), and PSP was distinguished from iPD (AUC = 0.758) and healthy controls (AUC = 0.88). Hence, miRNAs represent a promising alternative that could be integrated into diagnostic workflows.

Limited information is available regarding the biological roles of these dysregulated brain miRNAs. MiR-136-3p expression is upregulated in synaptoneurosomes during the preclinical stage of prion disease and has been shown to protect cells from neuroinflammation following ischemic insults [50]. MiR-153-3p enhances the neurogenesis of neural stem cells, improves the cognitive abilities of aged mice through the Notch signaling pathway [51], and regulates the expression of SNCA [20], APP [52], and SNAP-25, impacting motor neuron synaptic activity [53]. MiR-154-5p is downregulated in neuropathic pain, and its overexpression reduces inflammatory responses [54]. MiR-219a-5p is a necessary and sufficient regulator of oligodendrocyte development, myelination, and remyelination [55]. MiR-323a-3p is upregulated in major depressive disorder and mild cognitive impairment and is differentially regulated by 6-hydroxydopamine (6-OHDA) and ischemia/reperfusion injury [56]. MiR-329-5p and miR-495-3p are activity-regulated miRNAs essential for homeostatic synaptic depression in excitatory neurons [57]. The expression of miR-495-3p is upregulated following deep brain stimulation (DBS) [58]. MiR-330-5p targets mRNAs involved in activity-dependent synaptic plasticity in the hippocampus and protects neurons from amyloid β-protein production, oxidative stress, and mitochondrial dysfunction in Alzheimer’s disease (AD) [59]. MiR-382-5p targets the dopamine receptor D1 (DRD1), inhibiting DRD1-induced action potential responses [60]. MiR-409-3p expression is upregulated in reactive astrocytes and the hippocampal dentate gyrus of a rat model of depression [61]. Repetitive transcranial magnetic stimulation (rTMS), a non-invasive technique with demonstrated therapeutic effects in PD, decreases miR-409-3p levels [62]. MiR-433-3p targets follicle-stimulating hormone (FSH) expression [63] and is downregulated in AD [64]. MiR-487b-3p is downregulated in spinal cord injury and lipopolysaccharide-induced microglial cell models, and its overexpression inhibits cell inflammation and apoptosis [65]. MiR-3200-3p regulates the expression of CAMK2A, suggesting its involvement in Ras/Raf/MEK/ERK/CREB1 signaling [66].

Analysis of the genomic distribution of differentially expressed miRNAs revealed that over half clustered at the Chr14q32 locus (Fig. 4A). This included miR-136, miR-154, miR-323a, miR-329-1, miR-382, miR-409, miR-411, miR-487b, and miR-495. The coordinated dysregulation of these miRNAs suggests regulation by shared transcription factors or methylation patterns. Notably, the Chr14q32 region exhibits instability, with a reported 1.1 Mb micro-deletion (Chr14q32.2.q32.3) associated with motor delay, hypotonia, and feeding problems in some patients [67]. The clustered miRNAs have also been identified as highly stable over time, supporting their utility as biomarkers for neurodegenerative diseases [68]. These findings underscore the potential importance of the Chr14q32 miRNA cluster in the pathogenesis of parkinsonian syndromes.

The TransmiR v2 experimental, manually curated database was used to explore the TFs regulating the expression of these dysregulated brain miRNAs. This analysis revealed transcription binding sites for CREB1, CEBPB, and MAZ within half of the analyzed miRNAs. These TFs play crucial roles in neuronal survival, axonal growth, adult neurogenesis, synaptic plasticity, memory formation, and glial cell differentiation [69–71]. These findings suggest that epigenetic dysregulation at the Chr14q32 locus may be responsible for the differential expression of multiple neuronal miRNAs in Parkinsonism.

An in silico analysis using KEGG pathways investigated the molecular pathways affected by the dysregulated miRNAs. The most significantly dysregulated pathways included “Prion diseases,” “Hippo signaling pathway,” “Wnt signaling pathway,” “TGF-β signaling pathway,” “Ubiquitin-mediated proteolysis,” and “FoxO signaling pathway.” This broad spectrum of affected pathways suggests widespread systemic dysregulation in these syndromes. Parkinsonian syndromes are believed to exhibit, at least partly, prion-like pathogenesis due to the misfolding characteristics and intercellular spread of α-synuclein and TAU [72]. Ubiquitin-mediated proteolysis is critical for maintaining cellular proteostasis, and studies have shown reduced catalytic activities within the core 20/26S proteasome of dopaminergic neurons in PD patients [73]. The TGF-beta signaling pathway plays a crucial role in the development and survival of dopaminergic neurons [74]. The Wnt signaling pathway is also a key regulator of neurogenesis and is essential for developing and maintaining dopaminergic neurons [75]. It is impaired by aging, inflammation, oxidative stress, and genetic PD [76], and restoring signaling can promote neuronal rescue and regeneration in PD experimental models [77]. The FoxO family of transcription factors is crucial in stress resistance and apoptotic programs [78]. Overexpression of FOXO3 leads to acute apoptosis of dopaminergic neurons, while its downregulation protects these neurons from α-synuclein overexpression [79]. The Hippo signaling pathway is a pivotal tissue growth and development regulator. Overactive Hippo signaling intertwines with various neurodegenerative processes, including oxidative stress, chronic inflammation, compromised blood–brain barrier integrity, and mitochondrial dysfunction, leading to neuronal cell death [80].

Several limitations of this study should be acknowledged. First, the sample size is relatively small, particularly for MSA subgroups and PSP group, reflecting the rarity of these conditions and challenges in patient recruitment. This limitation is common in studies of atypical parkinsonian syndromes but necessitates validation in larger cohorts. Second, our focused approach examining brain-enriched miRNAs, while based on strong biological rationale, may have missed other potentially relevant plasma biomarkers. Additionally, longitudinal studies are needed to assess these miRNAs’ utility in monitoring early diagnosis and disease progression.

In summary, plasma brain-derived miRNAs were analyzed to identify biomarker signatures for subtyping parkinsonian syndromes. The miRNAs identified make up a robust set of dysregulated brain-associated miRNAs that can be further assessed, alongside other metrics, as diagnostic and therapeutic tools for parkinsonian syndromes. Importantly, existing knowledge of their neurological functions provides insights into the processes they regulate. In silico analysis offered a comprehensive overview of the pathways and processes they influence, enhancing the current understanding of their biological role. Additionally, Chr14q32 emerged as a hotspot for these dysregulated miRNAs. Crucially, they are co-regulated by specific transcription factors that govern neuronal survival, memory, and plasticity in the adult brain. Despite their clinical differences, the consistent expression patterns observed between MSA subtypes suggest these miRNAs may reflect fundamental disease mechanisms rather than clinical manifestations, warranting further investigation into their potential as early diagnostic markers.

## Supplementary Information

Below is the link to the electronic supplementary material.

Supplementary file1 (DOCX 141 KB)

## Data Availability

No datasets were generated or analysed during the current study.
