# Supplementary material for: Plasma miRNA Biomarker Signatures in Parkinsonian Syndromes
Source: Mol Neurobiol. 2025 Apr 4;62(8):10118–32. doi: 10.1007/s12035-025-04890-w (PMC12289787; doi:10.1007/s12035-025-04890-w)
Supplement: Supplementary file 1 — Supplementary file1 (DOCX 141 KB) [file 12035_2025_4890_MOESM1_ESM.docx]

**SUPPLEMENTARY FIGURE**

**Supplementary Figure 1. Correlation between miR-29a-3p expression and LEDD in the MSA-C subgroup.** The correlation is represented by Spearman's rank correlation coefficient (r) and its corresponding p-value. After Bonferroni correction for multiple comparisons, the significance threshold was set at p < 0.0017.

**
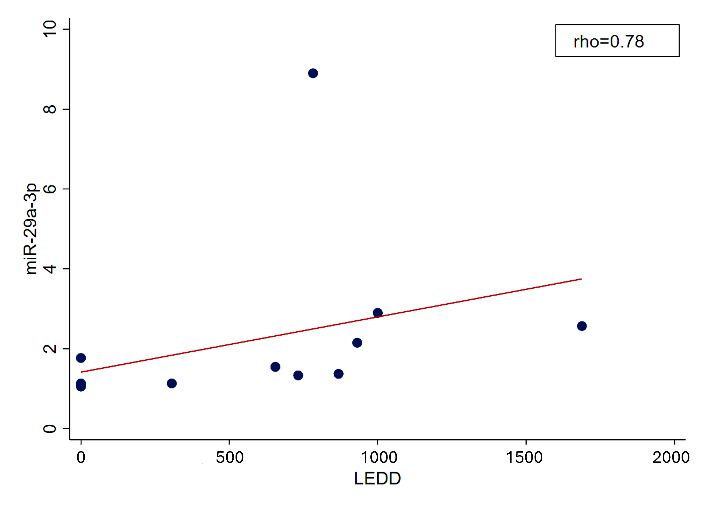
**

**SUPPLEMENTARY TABLES**

**Supplementary Table 1. Primers used for RT-qPCR.**

| **miRNA** | **Forward** | **Reverse** |
| --- | --- | --- |
| hsa-miR-7-5p | CGCAGTGGAAGACTAGTGA | GTCCAGTTTTTTTTTTTTTTTACAACA |
| hsa-miR-19b-3p | AGTGTGCAAATCCATGCA | CCAGTTTTTTTTTTTTTTTCAGTTTTG |
| hsa-miR-22-3p | GAAGCTGCCAGTTGAAGA | GTCCAGTTTTTTTTTTTTTTTACAGTT |
| hsa-miR-23a-3p | CATCACATTGCCAGGGAT | CGTCCAGTTTTTTTTTTTTTTTGGAA |
| hsa-miR-29a-3p | GCAGTAGCACCATCTGAAAT | TCCAGTTTTTTTTTTTTTTTAACCGA |
| hsa-miR-106a-5p | GAAAAGTGCTTACAGTGCAG | GGTCCAGTTTTTTTTTTTTTTTCTAC |
| hsa-miR-124-3p | CAGTAAGGCACGCGGTGA | GTCCAGTTTTTTTTTTTTTTTGGCATT |
| hsa-miR-127-3p | CGGATCCGTCTGAGCTT | TCCAGTTTTTTTTTTTTTTTAGCCA |
| hsa-miR-128-3p | AGTCACAGTGAACCGGTC | AGGTCCAGTTTTTTTTTTTTTTTAAAGA |
| hsa-miR-132-3p | GCAGTAACAGTCTACAGCCA | GTCCAGTTTTTTTTTTTTTTTCGAC |
| hsa-miR-136-3p | CGCAGCATCATCGTCTCA | GTCCAGTTTTTTTTTTTTTTTAGACTC |
| hsa-miR-153-3p | GCAGTTGCATAGTCACAAAAG | GTCCAGTTTTTTTTTTTTTTTGATCAC |
| hsa-miR-154-5p | CAGTAGGTTATCCGTGTTGC | GTCCAGTTTTTTTTTTTTTTTCGAAG |
| hsa-miR-191-5p | GCAACGGAATCCCAAAAG | CCAGTTTTTTTTTTTTTTTCAGCTG |
| hsa-miR-219a-5p | AGAATTGTGGCTGGACATC | GGTCCAGTTTTTTTTTTTTTTTACAG |
| hsa-miR-323a-3p | GCAGCACATTACACGGT | GTCCAGTTTTTTTTTTTTTTTAGAGGT |
| hsa-miR-329-5p | CGCAGAACACACCTGGT | GGTCCAGTTTTTTTTTTTTTTTAAAGAG |
| hsa-miR-330-5p | GTCTCTGGGCCTGTGT | CCAGTTTTTTTTTTTTTTTGCCTAAG |
| hsa-miR-338-3p | GCAGTCCAGCATCAGTGA | GGTCCAGTTTTTTTTTTTTTTTCAAC |
| hsa-miR-382-5p | CAGGAAGTTGTTCGTGGT | GTCCAGTTTTTTTTTTTTTTTCGAATC |
| hsa-miR-409-3p | AGGAATGTTGCTCGGTGA | CCAGTTTTTTTTTTTTTTTAGGGGTT |
| hsa-miR-410-3p | AGCGCAGAATATAACACAGATG | GGTCCAGTTTTTTTTTTTTTTTACAG |
| hsa-miR-411-5p | CAGTAGTAGACCGTATAGCGT | GGTCCAGTTTTTTTTTTTTTTTCGT |
| hsa-miR-423-3p | CGGTCTGAGGCCCCT | GGTCCAGTTTTTTTTTTTTTTTACTG |
| hsa-miR-425-5p | AGAATGACACGATCACTCC | TCCAGTTTTTTTTTTTTTTTCAACG |
| hsa-miR-432-5p | CAGTCTTGGAGTAGGTCATTG | GTCCAGTTTTTTTTTTTTTTTCCAC |
| hsa-miR-451a-5p | GCAGAAACCGTTACCATTAC | GTCCAGTTTTTTTTTTTTTTTAACTCA |
| hsa-miR-487b-3p | CAGAATCGTACAGGGTCATC | GGTCCAGTTTTTTTTTTTTTTTAAGTG |
| hsa-miR-495-3p | CGCAGAAACAAACATGGTG | GTCCAGTTTTTTTTTTTTTTTAAGAAGTG |
| hsa-miR-598-3p | CAGTACGTCATCGTTGTCATC | GGTCCAGTTTTTTTTTTTTTTTGAC |
| hsa-miR-654-3p | CAGTATGTCTGCTGACCATC | GTCCAGTTTTTTTTTTTTTTTAAGGTG |
| hsa-miR-885-5p | GCGCAGTCCATTACACTAC | AGGTCCAGTTTTTTTTTTTTTTTAGAG |
| hsa-miR-3200-3p | CACCTTGCGCTACTCAG | GTCCAGTTTTTTTTTTTTTTTCAGAC |
| miRNA RT | CAGGTCCAGTTTTTTTTTTTTTTTVN |  |

**Supplementary Table 2. Pairwise comparisons of plasma miRNA expression levels among iPD, MSA-C, MSA-P, PSP, and HC cohorts.** The expression levels of 28 miRNAs were compared between pairs of groups using Dunn's test with Benjamini-Hochberg correction for multiple comparisons.

| **miR-7-5p** | | | | | | | **miR-19b-3p** | | | | | | |
| --- | --- | --- | --- | --- | --- | --- | --- | --- | --- | --- | --- | --- | --- |
| KW | Chi2=8.979 | | Df=4 | | P=0.0616 | | KW | Chi2=6.223 | | Df=4 | | P=0.1830 | |
| Multiple Comparisons (Benjamini-Hochberg) | | | | | | | Multiple Comparisons (Benjamini-Hochberg) | | | | | | |
| p-values | MSA-C | MSA-P | | PSP | | iPD | p-values | MSA-C | MSA-P | | PSP | | iPD |
| MSA-P | 0.3474 |  | |  | |  | MSA-P | 0.3847 |  | |  | |  |
| PSP | 0.2719 | 0.2170 | |  | |  | PSP | 0.2265 | 0.2370 | |  | |  |
| iPD | 0.0907 | 0.0472 | | 0.2280 | |  | iPD | 0.1799 | 0.2191 | | 0.4273 | |  |
| CONTR | 0.1923 | 0.0862 | | 0.3214 | | 0.2997 | CONTR | 0.2984 | 0.3594 | | 0.2944 | | 0.1929 |
| **miR-22-3p** | | | | | | | **miR-29a-3p** | | | | | | |
| KW | Chi2=5.432 | | Df=4 | | P=0.2539 | | KW | Chi2=4.312 | | Df=4 | | P=0.3654 | |
| Multiple Comparisons (Benjamini-Hochberg) | | | | | | | Multiple Comparisons (Benjamini-Hochberg) | | | | | | |
| p-values | MSA-C | MSA-P | | PSP | | iPD | p-values | MSA-C | MSA-P | | PSP | | iPD |
| MSA-P | 0.5049 |  | |  | |  | MSA-P | 0.2712 |  | |  | |  |
| PSP | 0.2059 | 0.1384 | |  | |  | PSP | 0.5037 | 0.2181 | |  | |  |
| iPD | 0.5042 | 0.4571 | | 0.1377 | |  | iPD | 0.4206 | 0.2920 | | 0.4459 | |  |
| CONTR | 0.5401 | 0.5543 | | 0.1190 | | 0.5021 | CONTR | 0.4567 | 0.2613 | | 0.3759 | | 0.4295 |
| **miR-106a-5p** | | | | | | | **miR-124-3p** | | | | | | |
| KW | Chi2=7.363 | | Df=4 | | P=0.1179 | | KW | Chi2=8.430 | | Df=4 | | P=0.0770 | |
| Multiple Comparisons (Benjamini-Hochberg) | | | | | | | Multiple Comparisons (Benjamini-Hochberg) | | | | | | |
| p-values | MSA-C | MSA-P | | PSP | | iPD | p-values | MSA-C | MSA-P | | PSP | | iPD |
| MSA-P | 0.4880 |  | |  | |  | MSA-P | 0.2459 |  | |  | |  |
| PSP | 0.1420 | 0.1693 | |  | |  | PSP | 0.3182 | 0.1327 | |  | |  |
| iPD | 0.1560 | 0.0825 | | 0.3928 | |  | iPD | 0.2570 | 0.0540 | | 0.4179 | |  |
| CONTR | 0.2610 | 0.1968 | | 0.4616 | | 0.2660 | CONTR | 0.1666 | 0.0396 | | 0.3618 | | 0.3648 |
| **miR-127-3p** | | | | | | | **miR-128-3p** | | | | | | |
| KW | Chi2=2.564 | | Df=4 | | P=0.6332 | | KW | Chi2=3.988 | | Df=4 | | P=0.4076 | |
| Multiple Comparisons (Benjamini-Hochberg) | | | | | | | Multiple Comparisons (Benjamini-Hochberg) | | | | | | |
| p-values | MSA-C | MSA-P | | PSP | | iPD | p-values | MSA-C | MSA-P | | PSP | | iPD |
| MSA-P | 0.7110 |  | |  | |  | MSA-P | 0.2866 |  | |  | |  |
| PSP | 0.5284 | 0.4190 | |  | |  | PSP | 0.3689 | 0.3689 | |  | |  |
| iPD | 0.5359 | 0.4591 | | 0.4838 | |  | iPD | 0.3465 | 0.4854 | | 0.4628 | |  |
| CONTR | 0.4149 | 0.4742 | | 0.3984 | | 0.5052 | CONTR | 0.3618 | 0.3564 | | 0.3258 | | 0.3933 |
| **miR-132-3p** | | | | | | | **miR-136-3p** | | | | | | |
| KW | Chi2=4.301 | | Df=4 | | P=0.3667 | | KW | Chi2=16.838 | | Df=4 | | **P=0.0021** | |
| Multiple Comparisons (Benjamini-Hochberg) | | | | | | | Multiple Comparisons (Benjamini-Hochberg) | | | | | | |
| p-values | MSA-C | MSA-P | | PSP | | iPD | p-values | MSA-C | MSA-P | | PSP | | iPD |
| MSA-P | 0.4282 |  | |  | |  | MSA-P | 0.3333 |  | |  | |  |
| PSP | 0.2135 | 0.3227 | |  | |  | PSP | 0.1506 | 0.0842 | |  | |  |
| iPD | 0.2811 | 0.4100 | | 0.4459 | |  | iPD | **0.0080** | **0.0039** | | 0.1456 | |  |
| CONTR | 0.3063 | 0.2786 | | 0.3459 | | 0.3534 | CONTR | **0.0264** | **0.0103** | | 0.2815 | | 0.2736 |
| **miR-153-3p** | | | | | | | **miR-154-5p** | | | | | | |
| KW | Chi2=18.197 | | Df=4 | | **P=0.0011** | | KW | Chi2=24.656 | | Df=4 | | **P=0.0001** | |
| Multiple Comparisons (Benjamini-Hochberg) | | | | | | | Multiple Comparisons (Benjamini-Hochberg) | | | | | | |
| p-values | MSA-C | MSA-P | | PSP | | iPD | p-values | MSA-C | MSA-P | | PSP | | iPD |
| MSA-P | 0.4476 |  | |  | |  | MSA-P | 0.4344 |  | |  | |  |
| PSP | **0.0208** | **0.0221** | |  | |  | PSP | 0.1225 | 0.1112 | |  | |  |
| iPD | **0.0025** | **0.0041** | | 0.3854 | |  | iPD | **0.0004** | **0.0006** | | **0.0459** | |  |
| CONTR | **0.0483** | **0.0459** | | 0.2238 | | 0.0665 | CONTR | **0.0034** | **0.0032** | | 0.1245 | | 0.2010 |
| **miR-219a-5p** | | | | | | | **miR-323a-3p** | | | | | | |
| KW | Chi2=9.459 | | Df=4 | | P=0.0505 | | KW | Chi2=19.304 | | Df=4 | | **P=0.0007** | |
| Multiple Comparisons (Benjamini-Hochberg) | | | | | | | Multiple Comparisons (Benjamini-Hochberg) | | | | | | |
| p-values | MSA-C | MSA-P | | PSP | | iPD | p-values | MSA-C | MSA-P | | PSP | | iPD |
| MSA-P | 0.1614 |  | |  | |  | MSA-P | 0.4670 |  | |  | |  |
| PSP | 0.0561 | 0.3778 | |  | |  | PSP | **0.0296** | **0.0435** | |  | |  |
| iPD | 0.0155 | 0.2917 | | 0.4759 | |  | iPD | **0.0014** | **0.0023** | | 0.2050 | |  |
| CONTR | 0.0302 | 0.3454 | | 0.5310 | | 0.4955 | CONTR | **0.0203** | **0.0337** | | 0.4628 | | 0.1218 |
| **miR-329-5p** | | | | | | | **miR-330-3p** | | | | | | |
| KW | Chi2=18.044 | | Df=4 | | **P=0.0012** | | KW | Chi2=15.106 | | Df=4 | | **P=0.0045** | |
| Multiple Comparisons (Benjamini-Hochberg) | | | | | | | Multiple Comparisons (Benjamini-Hochberg) | | | | | | |
| p-values | MSA-C | MSA-P | | PSP | | iPD | p-values | MSA-C | MSA-P | | PSP | | iPD |
| MSA-P | 0.3045 |  | |  | |  | MSA-P | 0.1981 |  | |  | |  |
| PSP | 0.0945 | **0.0205** | |  | |  | PSP | **0.0131** | 0.0634 | |  | |  |
| iPD | **0.0115** | **0.0020** | | 0.2795 | |  | iPD | **0.0032** | **0.0384** | | 0.4742 | |  |
| CONTR | **0.0255** | **0.0039** | | 0.3448 | | 0.3580 | CONTR | **0.0329** | 0.1901 | | 0.1800 | | 0.1245 |
| **miR-338-3p** | | | | | | | **miR-382-5p** | | | | | | |
| KW | Chi2=10.913 | | Df=4 | | **P=0.0275** | | KW | Chi2=23.982 | | Df=4 | | **P=0.0001** | |
| Multiple Comparisons (Benjamini-Hochberg) | | | | | | | Multiple Comparisons (Benjamini-Hochberg) | | | | | | |
| p-values | MSA-C | MSA-P | | PSP | | iPD | p-values | MSA-C | MSA-P | | PSP | | iPD |
| MSA-P | 0.3089 |  | |  | |  | MSA-P | 0.2725 |  | |  | |  |
| PSP | 0.2937 | 0.4826 | |  | |  | PSP | 0.1168 | 0.0514 | |  | |  |
| iPD | **0.0263** | 0.1140 | | 0.0861 | |  | iPD | **0.0012** | **0.0002** | | 0.0704 | |  |
| CONTR | **0.0446** | 0.1317 | | 0.1554 | | 0.3390 | CONTR | **0.0115** | **0.0021** | | 0.2094 | | 0.1762 |
| **miR-409-3p** | | | | | | | **miR-410-3p** | | | | | | |
| KW | Chi2=20.031 | | Df=4 | | **P=0.0005** | | KW | Chi2=10.001 | | Df=4 | | **P=0.0404** | |
| Multiple Comparisons (Benjamini-Hochberg) | | | | | | | Multiple Comparisons (Benjamini-Hochberg) | | | | | | |
| p-values | MSA-C | MSA-P | | PSP | | iPD | p-values | MSA-C | MSA-P | | PSP | | iPD |
| MSA-P | 0.4946 |  | |  | |  | MSA-P | 0.1295 |  | |  | |  |
| PSP | **0.0212** | **0.0201** | |  | |  | PSP | 0.3504 | 0.1571 | |  | |  |
| iPD | **0.0015** | **0.0023** | | 0.2965 | |  | iPD | **0.0263** | 0.3002 | | 0.0692 | |  |
| CONTR | **0.0142** | **0.0145** | | 0.4455 | | 0.1968 | CONTR | 0.1343 | 0.3652 | | 0.1981 | | 0.1535 |
| **miR-411-5p** | | | | | | | **miR-432-5p** | | | | | | |
| KW | Chi2=18.569 | | Df=4 | | **P=0.0010** | | KW | Chi2=11.326 | | Df=4 | | **P=0.0231** | |
| Multiple Comparisons (Benjamini-Hochberg) | | | | | | | Multiple Comparisons (Benjamini-Hochberg) | | | | | | |
| p-values | MSA-C | MSA-P | | PSP | | iPD | p-values | MSA-C | MSA-P | | PSP | | iPD |
| MSA-P | 0.4143 |  | |  | |  | MSA-P | 0.3668 |  | |  | |  |
| PSP | **0.0114** | **0.0079** | |  | |  | PSP | 0.0501 | **0.0371** | |  | |  |
| iPD | **0.0082** | **0.0062** | | 0.4948 | |  | iPD | **0.0482** | **0.0429** | | 0.3973 | |  |
| CONTR | **0.0109** | **0.0073** | | 0.4387 | | 0.4544 | CONTR | 0.1102 | **0.0492** | | 0.2620 | | 0.2572 |
| **miR-487b-3p** | | | | | | | **miR-495-3p** | | | | | | |
| KW | Chi2=18.089 | | Df=4 | | **P=0.0012** | | KW | Chi2=15.258 | | Df=4 | | **P=0.0042** | |
| Multiple Comparisons (Benjamini-Hochberg) | | | | | | | Multiple Comparisons (Benjamini-Hochberg) | | | | | | |
| p-values | MSA-C | MSA-P | | PSP | | iPD | p-values | MSA-C | MSA-P | | PSP | | iPD |
| MSA-P | 0.4429 |  | |  | |  | MSA-P | 0.4861 |  | |  | |  |
| PSP | **0.0180** | **0.0196** | |  | |  | PSP | 0.0647 | 0.0626 | |  | |  |
| iPD | **0.0046** | **0.0055** | | 0.4495 | |  | iPD | **0.0070** | **0.0054** | | 0.2214 | |  |
| CONTR | **0.0124** | **0.0146** | | 0.4490 | | 0.3151 | CONTR | 0.0795 | 0.0737 | | 0.4587 | | 0.1017 |
| **miR-598-3p** | | | | | | | **miR-654-3p** | | | | | | |
| KW | Chi2=3.735 | | Df=4 | | P=0.4429 | | KW | Chi2=2.030 | | Df=4 | | P=0.7302 | |
| Multiple Comparisons (Benjamini-Hochberg) | | | | | | | Multiple Comparisons (Benjamini-Hochberg) | | | | | | |
| p-values | MSA-C | MSA-P | | PSP | | iPD | p-values | MSA-C | MSA-P | | PSP | | iPD |
| MSA-P | 0.4182 |  | |  | |  | MSA-P | 0.4981 |  | |  | |  |
| PSP | 0.3663 | 0.4819 | |  | |  | PSP | 0.4781 | 0.4707 | |  | |  |
| iPD | 0.4827 | 0.6587 | | 0.3398 | |  | iPD | 0.9999 | 0.6663 | | 0.7463 | |  |
| CONTR | 0.4319 | 0.4611 | | 0.4473 | | 0.3636 | CONTR | 0.5762 | 0.4794 | | 0.5258 | | 0.5036 |
| **miR-885-5p** | | | | | | | **miR-3200-39** | | | | | | |
| KW | Chi2=5.256 | | Df=4 | | P=0.1808 | | KW | Chi2=12.447 | | Df=4 | | **P=0.0143** | |
| Multiple Comparisons (Benjamini-Hochberg) | | | | | | | Multiple Comparisons (Benjamini-Hochberg) | | | | | | |
| p-values | MSA-C | MSA-P | | PSP | | iPD | p-values | MSA-C | MSA-P | | PSP | | iPD |
| MSA-P | 0.2563 |  | |  | |  | MSA-P | 0.4113 |  | |  | |  |
| PSP | 0.2479 | 0.1251 | |  | |  | PSP | 0.2940 | 0.2541 | |  | |  |
| iPD | 0.2783 | 0.1726 | | 0.2574 | |  | iPD | **0.0144** | **0.0163** | | 0.0916 | |  |
| CONTR | 0.4131 | 0.2446 | | 0.1602 | | 0.2806 | CONTR | 0.2018 | 0.1633 | | 0.4297 | | 0.0908 |

*Shapiro-Wilk test for normality revealed that most miRNAs were not normally distributed. Consequently, the Kruskal-Wallis test (non-parametric) was employed to evaluate the null hypothesis that each miRNA's distribution is identical across patient groups (MSA-C, MSA-P, PSP, iPD, and Control). Following the Kruskal-Wallis test, multiple comparisons were conducted using Dunn's test with Benjamini-Hochberg correction to identify significant differences between specific groups.*

**Supplementary Table 3. Association of plasma miRNA expression with clinical and demographic variables in each cohort.** The relative expression of each miRNA was correlated with age, age at onset, iPD duration, UPDRS III score, and LEDD. Spearman's rank correlation coefficient was employed to assess the strength and direction of these associations, with Bonferroni correction applied to account for multiple comparisons. This non-parametric approach was chosen due to the non-normal distribution of miRNA expression. The adjusted significance threshold was set at p < 0.0017.

| **MSA-C** | **Age** | **Age at onset** | **iPD duration** | **UPDRS III** | **LEDD** |
| --- | --- | --- | --- | --- | --- |
|  | Rho (p-value) | Rho (p-value) | Rho (p-value) | Rho (p-value) | Rho (p-value) |
| miR-7-5p | 0.2469 (0.4161) | 0.3802 (0.2001) | -0.0474 (0.8779) | 0.2083 (0.4947) | 0.6254 (0.0223) |
| miR-19b-3p | 0.3361 (0.2615) | 0.4182 (0.1550) | 0.0083 (0.9784) | 0.1791 (0.5583) | 0.6782 (0.0108) |
| miR-22-3p | 0.2469 (0.4161) | 0.3402 (0.2554) | -0.1100 (0.7205) | 0.2110 (0.4889) | 0.5462 (0.0535) |
| miR-29a-3p | 0.2314 (0.4468) | 0.2338 (0.4419) | 0.2448 (0.4202) | 0.4766 (0.0997) | **0.7800 (0.0017) *** |
| miR-106a-5p | 0.1433 (0.6406) | 0.2311 (0.4475) | 0.0640 (0.8355) | 0.1405 (0.6471) | 0.6217 (0.0233) |
| miR-124-3p | -0.1025 (0.7390) | 0.0387 (0.9000) | -0.1678 (0.5836) | -0.0305 (0.9213) | 0.3978 (0.1782) |
| miR-127-3p | -0.0523 (0.8652) | -0.0963 (0.7543) | 0.0417 (0.8923) | 0.1019 (0.7404) | -0.0113 (0.9708) |
| miR-128-3p | -0.2149 (0.4808) | -0.0963 (0.7543) | -0.0139 (0.9640) | -0.2975 (0.3235) | 0.0283 (0.9270) |
| miR-132-3p | -0.0138 (0.9644) | 0.0138 (0.9644) | 0.3255 (0.2778) | 0.1598 (0.6021) | 0.4295 (0.1430) |
| miR-136-3p | -0.2066 (0.4982) | -0.3384 (0.2581) | 0.2726 (0.3675) | -0.3939 (0.1829) | -0.2826 (0.3495) |
| miR-153-3p | -0.6997 (0.0078) | -0.6905 (0.0090) | 0.1780 (0.5606) | -0.2590 (0.3929) | -0.1809 (0.5543) |
| miR-154-5p | -0.4490 (0.1237) | -0.6190 (0.0241) | 0.5842 (0.0360) | -0.0661 (0.8301) | -0.0735 (0.8115) |
| miR-219a-5p | 0.4155 (0.1579) | 0.5007 (0.0814) | -0.4615 (0.1124) | 0.0471 (0.8786) | 0.0511 (0.8682) |
| miR-323a-3p | -0.0661 (0.8301) | -0.1843 (0.5466) | 0.1447 (0.6373) | -0.1129 (0.7133) | -0.2883 (0.3395) |
| miR-329-5p | -0.5545 (0.0492) | -0.6501 (0.0161) | 0.1797 (0.5570) | -0.3586 (0.2289) | -0.5971 (0.0312) |
| miR-330-3p | -0.0826 (0.7884) | -0.2889 (0.3385) | 0.5564 (0.0483) | 0.2452 (0.4195) | 0.0678 (0.8258) |
| miR-338-3p | -0.0358 (0.9075) | -0.0825 (0.7887) | 0.2392 (0.4312) | 0.3581 (0.2296) | 0.3504 (0.2405) |
| miR-382-5p | -0.3526 (0.2373) | -0.4567 (0.1167) | 0.2059 (0.4998) | -0.3141 (0.2960) | -0.4974 (0.0837) |
| miR-409-3p | -0.2837 (0.3475) | -0.4319 (0.1405) | 0.3561 (0.2324) | -0.1405 (0.6471) | -0.2769 (0.3597) |
| miR-410-3p | -0.5840 (0.0361) | -0.5282 (0.0635) | 0.0946 (0.7586) | -0.2948 (0.3282) | -0.1922 (0.5294) |
| miR-411-5p | 0.2428 (0.4242) | 0.0441 (0.8863) | 0.4429 (0.1296) | -0.0441 (0.8862) | 0.0566 (0.8543) |
| miR-432-5p | -0.0964 (0.7540) | -0.2366 (0.4364) | 0.4173 (0.1560) | -0.1377 (0.6536) | -0.1583 (0.6056) |
| miR-487b-3p | 0.0496 (0.8722) | -0.0633 (0.8373) | 0.0501 (0.8710) | -0.0523 (0.8652) | -0.3448 (0.2486) |
| miR-495-3p | -0.4050 (0.1699) | -0.4264 (0.1462) | -0.0612 (0.8426) | -0.4077 (0.1667) | -0.6161 (0.0250) |
| miR-598-3p | 0.1867 (0.5413) | 0.3191 (0.2880) | -0.1411 (0.6457) | -0.1342 (0.6621) | 0.0341 (0.9121) |
| miR-654-3p | -0.2979 (0.3228) | -0.3140 (0.2960) | 0.4304 (0.1421) | 0.1476 (0.6304) | 0.3056 (0.3099) |
| miR-885-5p | 0.2041 (0.5035) | 0.2672 (0.3775) | 0.0669 (0.8282) | 0.4234 (0.1494) | 0.6792 (0.0107) |
| miR-3200-3p | 0.1488 (0.6277) | 0.2669 (0.3781) | -0.2225 (0.4649) | 0.2810 (0.3524) | 0.4522 (0.1208) |
| **MSA-P** | **Age** | **Age at onset** | **iPD duration** | **UPDRS III** | **LEDD** |
|  | Rho (p-value) | Rho (p-value) | Rho (p-value) | Rho (p-value) | Rho (p-value) |
| miR-7-5p | 0.2657 (0.4038) | 0.3222 (0.3070) | 0.0438 (0.8924) | -0.1158 (0.7201) | 0.1786 (0.5786) |
| miR-19b-3p | 0.0839 (0.7954) | 0.1786 (0.5786) | -0.0438 (0.8924) | -0.3053 (0.3346) | 0.2277 (0.4767) |
| miR-22-3p | 0.2378 (0.4568) | 0.1121 (0.7287) | 0.3361 (0.2855) | 0.1544 (0.6319) | -0.0560 (0.8627) |
| miR-29a-3p | -0.2657 (0.4038) | -0.1926 (0.5486) | 0.0146 (0.9640) | -0.4807 (0.1137) | 0.6235 (0.0303) |
| miR-106a-5p | 0.2937 (0.3541) | 0.3117 (0.3239) | 0.0658 (0.8391) | 0.1088 (0.7365) | 0.0280 (0.9311) |
| miR-124-3p | -0.2378 (0.4568) | -0.1331 (0.6801) | 0.2046 (0.5236) | -0.2982 (0.3464) | 0.7426 (0.0057) |
| miR-127-3p | 0.3916 (0.2081) | 0.3363 (0.2852) | -0.1754 (0.5857) | 0.3228 (0.3061) | -0.4063 (0.1900) |
| miR-128-3p | -0.0839 (0.7954) | -0.2802 (0.3777) | 0.5261 (0.0790) | 0.1158 (0.7201) | -0.1401 (0.6641) |
| miR-132-3p | -0.0070 (0.9828) | -0.0806 (0.8035) | 0.2338 (0.4646) | -0.1719 (0.5931) | 0.1541 (0.6325) |
| miR-136-3p | 0.0140 (0.9656) | 0.0175 (0.9569) | -0.1461 (0.6504) | -0.2912 (0.3584) | -0.1436 (0.6561) |
| miR-153-3p | 0.0839 (0.7954) | -0.0630 (0.8457) | 0.0950 (0.7690) | 0.3088 (0.3288) | -0.2487 (0.4357) |
| miR-154-5p | 0.5039 (0.0952) | 0.4623 (0.1302) | -0.1607 (0.6177) | 0.1544 (0.6319) | -0.4448 (0.1473) |
| miR-219a-5p | -0.2937 (0.3541) | -0.0876 (0.7867) | -0.0804 (0.8039) | -0.3579 (0.2534) | 0.2067 (0.5193) |
| miR-323a-3p | 0.0559 (0.8629) | -0.0455 (0.8883) | -0.1534 (0.6340) | 0.1684 (0.6008) | -0.6060 (0.0368) |
| miR-329-5p | -0.1339 (0.6646) | -0.2382 (0.4560) | 0.1242 (0.7005) | 0.3158 (0.3173) | -0.1051 (0.7452) |
| miR-330-3p | 0.0736 (0.8203) | -0.0877 (0.7863) | 0.0805 (0.8036) | -0.2531 (0.4274) | -0.0649 (0.8411) |
| miR-338-3p | 0.2657 (0.4038) | 0.0911 (0.7783) | 0.1534 (0.6340) | 0.2912 (0.3584) | -0.2697 (0.3966) |
| miR-382-5p | 0.3187 (0.3126) | 0.0649 (0.8411) | 0.3952 (0.2035) | 0.2091 (0.5142) | 0.2807 (0.3768) |
| miR-409-3p | -0.0806 (0.8035) | -0.2088 (0.5149) | -0.0512 (0.8744) | 0.1652 (0.6079) | -0.5684 (0.0538) |
| miR-410-3p | -0.1538 (0.6331) | -0.1436 (0.6561) | 0.2046 (0.5236) | -0.0526 (0.8710) | 0.5149 (0.0867) |
| miR-411-5p | 0.2737 (0.3894) | 0.2671 (0.4013) | 0.0000 (>0.9999) | 0.0475 (0.8834) | 0.0527 (0.8707) |
| miR-432-5p | 0.0909 (0.7787) | 0.0560 (0.8627) | -0.0438 (0.8924) | -0.1474 (0.6476) | -0.3888 (0.2116) |
| miR-487b-3p | 0.2587 (0.4168) | 0.2907 (0.3593) | -0.2411 (0.4503) | 0.2246 (0.4829) | -0.3923 (0.2072) |
| miR-495-3p | 0.3147 (0.3191) | 0.2662 (0.4030) | -0.1680 (0.6016) | -0.0561 (0.8624) | -0.5639 (0.0562) |
| miR-598-3p | 0.1888 (0.5567) | 0.1191 (0.7124) | 0.0365 (0.9103) | 0.0140 (0.9655) | -0.3538 (0.2593) |
| miR-654-3p | 0.3287 (0.2969) | 0.3012 (0.3414) | 0.1315 (0.6837) | 0.1018 (0.7530) | -0.0315 (0.9225) |
| miR-885-5p | -0.1399 (0.6646) | -0.1156 (0.7206) | 0.0073 (0.9820) | -0.3404 (0.2790) | 0.7461 (0.0053) |
| miR-3200-3p | -0.2028 (0.5273) | -0.1366 (0.6721) | 0.0877 (0.7864) | -0.2386 (0.4552) | 0.4659 (0.1269) |
| **PSP** | **Age** | **Age at onset** | **iPD duration** | **UPDRS III** | **LEDD** |
|  | Rho (p-value) | Rho (p-value) | Rho (p-value) | Rho (p-value) | Rho (p-value) |
| miR-7-5p | -0.2727 (0.4171) | -0.0228 (0.9470) | -0.4441 (0.1712) | -0.5091 (0.1097) | -0.1139 (0.7388) |
| miR-19b-3p | -0.2182 (0.5192) | 0.0273 (0.9364) | -0.1496 (0.6607) | -0.3455 (0.2981) | -0.0137 (0.9682) |
| miR-22-3p | 0.5455 (0.0827) | 0.5604 (0.0730) | 0.1356 (0.6910) | 0.0545 (0.8734) | 0.3462 (0.2969) |
| miR-29a-3p | -0.0909 (0.7904) | 0.0410 (0.9047) | -0.3646 (0.2702) | -0.6636 (0.0260) | -0.2278 (0.5005) |
| miR-106a-5p | 0.0636 (0.8525) | 0.2415 (0.4744) | -0.1122 (0.7426) | -0.3364 (0.3118) | -0.0410 (0.9047) |
| miR-124-3p | 0.0909 (0.7904) | 0.1595 (0.6396) | -0.4675 (0.1471) | -0.8182 (0.0021) | -0.4237 (0.1941) |
| miR-127-3p | -0.1455 (0.6696) | -0.1321 (0.6986) | -0.0187 (0.9565) | -0.1182 (0.7293) | -0.3189 (0.3391) |
| miR-128-3p | 0.2455 (0.4669) | 0.1002 (0.7694) | 0.4348 (0.1815) | -0.2545 (0.4500) | 0.1367 (0.6886) |
| miR-132-3p | 0.0411 (0.9045) | 0.1373 (0.6873) | 0.2090 (0.5374) | -0.1553 (0.6485) | 0.3066 (0.3591) |
| miR-136-3p | -0.1636 (0.6307) | -0.1595 (0.6396) | 0.0234 (0.9456) | 0.1091 (0.7495) | -0.1276 (0.7086) |
| miR-153-3p | 0.3909 (0.2345) | 0.2460 (0.4659) | 0.2618 (0.4368) | 0.3182 (0.3403) | 0.2597 (0.4406) |
| miR-154-5p | -0.2364 (0.4841) | -0.1822 (0.5918) | -0.1262 (0.7115) | 0.2091 (0.5372) | -0.2232 (0.5094) |
| miR-219a-5p | -0.2455 (0.4669) | -0.2779 (0.4080) | 0.0047 (0.9891) | -0.1091 (0.7495) | 0.0000 (>0.999) |
| miR-323a-3p | -0.4091 (0.2115) | -0.3554 (0.2835) | -0.1075 (0.7530) | 0.4909 (0.1252) | -0.2232 (0.5094) |
| miR-329-5p | 0.0273 (0.9366) | -0.0319 (0.9258) | 0.2571 (0.4453) | 0.4727 (0.1420) | -0.0137 (0.9682) |
| miR-330-3p | -0.0911 (0.7899) | -0.3447 (0.2991) | 0.3748 (0.2560) | -0.2141 (0.5272) | -0.0457 (0.8939) |
| miR-338-3p | 0.0273 (0.9366) | -0.2096 (0.5363) | 0.3880 (0.2383) | -0.2364 (0.4841) | 0.2050 (0.5454) |
| miR-382-5p | -0.0455 (0.8944) | -0.2050 (0.5454) | 0.1730 (0.6110) | 0.5727 (0.0655) | -0.1913 (0.5730) |
| miR-409-3p | -0.2091 (0.5372) | -0.1321 (0.6986) | -0.2852 (0.3953) | 0.4455 (0.1697) | -0.5740 (0.0648) |
| miR-410-3p | 0.3273 (0.3259) | 0.2187 (0.5183) | 0.1496 (0.6607) | -0.2182 (0.5192) | -0.1503 (0.6590) |
| miR-411-5p | -0.2545 (0.4500) | -0.1686 (0.6203) | -0.0187 (0.9565) | 0.5364 (0.0890) | -0.2096 (0.5363) |
| miR-432-5p | -0.0636 (0.8525) | -0.0866 (0.8002) | 0.0935 (0.7845) | 0.5000 (0.1173) | -0.2369 (0.4831) |
| miR-487b-3p | 0.0000 (>0.9999) | 0.0228 (0.9470) | -0.1589 (0.6407) | 0.1636 (0.6307) | -0.5103 (0.1088) |
| miR-495-3p | 0.0273 (0.9366) | 0.0000 (>0.999) | -0.0561 (0.8699) | 0.0909 (0.7904) | -0.4647 (0.1498) |
| miR-598-3p | 0.3000 (0.3701) | 0.3326 (0.3176) | 0.5002 (0.1171) | 0.4182 (0.2006) | 0.5877 (0.0573) |
| miR-654-3p | -0.2460 (0.4659) | -0.3425 (0.3026) | -0.0164 (0.9618) | 0.3326 (0.3176) | -0.4247 (0.1930) |
| miR-885-5p | 0.0137 (0.9681) | 0.2060 (0.5435) | -0.5612 (0.0725) | -0.4247 (0.1930) | -0.0046 (0.9893) |
| miR-3200-3p | -0.3909 (0.2345) | -0.1549 (0.6493) | -0.5095 (0.1094) | -0.6818 (0.0208) | -0.2323 (0.4918) |
| **iPD** | **Age** | **Age at onset** | **iPD duration** | **UPDRS III** | **LEDD** |
|  | Rho (p-value) | Rho (p-value) | Rho (p-value) | Rho (p-value) | Rho (p-value) |
| miR-7-5p | -0.1808 (0.3870) | -0.0949 (0.6519) | 0.1222 (0.5608) | 0.1983 (0.3421) | 0.0187 (0.9293) |
| miR-19b-3p | -0.1750 (0.4027) | -0.0470 (0.8233) | 0.1254 (0.5502) | -0.0789 (0.7077) | 0.0158 (0.9402) |
| miR-22-3p | -0.1718 (0.4116) | -0.1437 (0.4932) | 0.1656 (0.4288) | 0.1130 (0.5907) | 0.0891 (0.6720) |
| miR-29a-3p | -0.3145 (0.1258) | 0.0027 (0.9898) | -0.1625 (0.4378) | 0.1436 (0.4936) | 0.0728 (0.7293) |
| miR-106a-5p | -0.2113 (0.3106) | -0.3289 (0.1084) | 0.2548 (0.2190) | -0.1132 (0.5902) | 0.1368 (0.5143) |
| miR-124-3p | -0.0572 (0.7860) | 0.2006 (0.3364) | -0.2744 (0.1844) | 0.2661 (0.1986) | 0.0008 (0.9971) |
| miR-127-3p | 0.0715 (0.7341) | -0.1470 (0.4833) | 0.1845 (0.3772) | 0.0037 (0.9862) | 0.1939 (0.3529) |
| miR-128-3p | -0.1607 (0.4428) | -0.1882 (0.3677) | 0.0951 (0.6513) | -0.4199 (0.0366) | -0.0732 (-0.0732) |
| miR-132-3p | -0.2202 (0.2901) | -0.1897 (0.3637) | 0.1492 (0.4765) | 0.2033 (0.3298) | -0.1037 (0.6218) |
| miR-136-3p | -0.0359 (0.8646) | -0.2020 (0.3328) | 0.1753 (0.4019) | 0.0535 (0.7995) | 0.2120 (0.3090) |
| miR-153-3p | -0.0209 (0.9212) | -0.0085 (0.9679) | -0.1305 (0.5341) | 0.0416 (0.8436) | -0.1438 (0.4930) |
| miR-154-5p | -0.0568 (0.7874) | -0.2024 (0.3318) | 0.1247 (0.5527) | 0.1220 (0.5612) | 0.1611 (0.4417) |
| miR-219a-5p | -0.0184 (0.9306) | 0.0183 (0.9307) | -0.0279 (0.8948) | -0.0697 (0.7407) | -0.0359 (0.8649) |
| miR-323a-3p | -0.1024 (0.6263) | -0.1920 (0.3578) | 0.0779 (0.7112) | 0.0727 (0.7297) | 0.1114 (0.5960) |
| miR-329-5p | 0.0425 (0.8402) | -0.2017 (0.3337) | 0.1812 (0.3861) | -0.0158 (0.9403) | 0.1985 (0.3415) |
| miR-330-3p | 0.0583 (0.7818) | -0.1039 (0.6210) | 0.1602 (0.4444) | 0.1105 (0.5990) | 0.0281 (0.8938) |
| miR-338-3p | -0.0926 (0.6599) | -0.2048 (0.3260) | 0.2557 (0.2174) | 0.1588 (0.4482) | 0.1806 (0.3875) |
| miR-382-5p | -0.0093 (0.9649) | -0.1943 (0.3519) | 0.1192 (0.5703) | 0.1632 (0.4357) | 0.1696 (0.4177) |
| miR-409-3p | 0.0106 (0.9598) | -0.1980 (0.3427) | 0.1284 (0.5408) | 0.0435 (0.8364) | 0.1390 (0.5076) |
| miR-410-3p | -0.1518 (0.4688) | -0.0416 (0.8433) | 0.1239 (0.5552) | -0.1501 (0.4739) | 0.0605 (0.7739) |
| miR-411-5p | 0.0388 (0.8538) | -0.1886 (0.3666) | 0.1270 (0.5451) | 0.2054 (0.3247) | 0.1768 (0.3980) |
| miR-432-5p | 0.0236 (0.9110) | -0.2036 (0.3290) | 0.1613 (0.4412) | 0.1694 (0.4183) | 0.2386 (0.2508) |
| miR-487b-3p | -0.0394 (0.8517) | -0.2518 (0.2247) | 0.1204 (0.5665) | 0.1005 (0.6328) | 0.1761 (0.3997) |
| miR-495-3p | 0.0807 (0.7012) | -0.0224 (0.9155) | -0.0771 (0.7140) | 0.1859 (0.3736) | 0.0917 (0.6628) |
| miR-598-3p | -0.4624 (0.0199) | -0.2151 (0.3017) | -0.0868 (0.6801) | -0.3211 (0.1176) | -0.1134 (0.5894) |
| miR-654-3p | -0.0537 (0.7988) | 0.1943 (0.3519) | -0.4574 (0.0215) | -0.1520 (0.4681) | -0.1376 (0.5119) |
| miR-885-5p | -0.1832 (0.3808) | 0.0486 (0.8176) | -0.0503 (0.8114) | 0.2604 (0.2086) | -0.0042 (0.9839) |
| miR-3200-3p | -0.2114 (0.3105) | -0.1054 (0.6161) | 0.1874 (0.3697) | 0.0489 (0.8163) | 0.0328 (0.8763) |
| **CONTROL** | **Age** |  |  |  |  |
|  | Rho (p-value) |  |  |  |  |
| miR-7-5p | -0.0052 (0.9803) |  |  |  |  |
| miR-19b-3p | -0.1087 (0.6050) |  |  |  |  |
| miR-22-3p | 0.0137 (0.9482) |  |  |  |  |
| miR-29a-3p | -0.0744 (0.7238) |  |  |  |  |
| miR-106a-5p | 0.1673 (0.4242) |  |  |  |  |
| miR-124-3p | 0.0731 (0.7285) |  |  |  |  |
| miR-127-3p | -0.0382 (0.8563) |  |  |  |  |
| miR-128-3p | 0.0782 (0.7101) |  |  |  |  |
| miR-132-3p | -0.2379 (0.2522) |  |  |  |  |
| miR-136-3p | 0.0921 (0.6614) |  |  |  |  |
| miR-153-3p | 0.0584 (0.7815) |  |  |  |  |
| miR-154-5p | 0.0952 (0.6508) |  |  |  |  |
| miR-219a-5p | -0.2149 (0.3022) |  |  |  |  |
| miR-323a-3p | 0.1058 (0.6146) |  |  |  |  |
| miR-329-5p | 0.1314 (0.5312) |  |  |  |  |
| miR-330-3p | 0.1315 (0.5311) |  |  |  |  |
| miR-338-3p | -0.3310 (0.1060) |  |  |  |  |
| miR-382-5p | 0.2635 (0.2032) |  |  |  |  |
| miR-409-3p | 0.2067 (0.3216) |  |  |  |  |
| miR-410-3p | 0.2991 (0.1464) |  |  |  |  |
| miR-411-5p | 0.1792 (0.3913) |  |  |  |  |
| miR-432-5p | 0.1199 (0.5682) |  |  |  |  |
| miR-487b-3p | 0.0879 (0.6762) |  |  |  |  |
| miR-495-3p | 0.1503 (0.4732) |  |  |  |  |
| miR-598-3p | 0.0366 (0.8620) |  |  |  |  |
| miR-654-3p | -0.0468 (0.8241) |  |  |  |  |
| miR-885-5p | 0.1295 (0.5372) |  |  |  |  |
| miR-3200-3p | -0.1945 (0.3515) |  |  |  |  |

**Supplementary Table 4. Association of plasma miRNA with cognitive and motor assessment scores in each cohort.** The relative expression of each miRNA was correlated with the following variables: MoCA, FAB, Digit Span (Total, Fw, Bw), Goldenberg Scale, PSP score, UMSARS Total, SCOPA Total, H&Y stage, and autonomic function measures (subSBP, subDBP, 30:15 ratio, DBP change in HR, DBP difference in hand grip, Valsalva ratio). Spearman's rank correlation coefficient was employed to assess the strength and direction of these associations, with Bonferroni correction applied to account for multiple comparisons. This non-parametric approach was chosen due to the non-normal distribution of miRNA expression. The adjusted significance threshold was set at p < 0.0017.

|  | **MOCA** | **FAB** | **Goldenberg Scale** | **PSP score** |
| --- | --- | --- | --- | --- |
|  | Rho (p-value) | Rho (p-value) | Rho (p-value) | Rho (p-value) |
| miR-7-5p | -0.0226 (0.8625) | 0.0878 (0.5012) | 0.1858 (0.2779) | -0.1777 (0.2998) |
| miR-19b-3p | 0.1164 (0.3717) | 0.0848 (0.5161) | 0.1792 (0.2956) | -0.2356 (0.1667) |
| miR-22-3p | 0.0084 (0.9487) | -0.0772 (0.5540) | 0.1290 (0.4535) | 0.0355 (0.8372) |
| miR-29a-3p | 0.2195 (0.0892) | 0.1113 (0.3933) | 0.1718 (0.3165) | -0.1226 (0.4762) |
| miR-106a-5p | 0.0656 (0.6153) | 0.1205 (0.3551) | 0.1805 (0.2921) | -0.1726 (0.3142) |
| miR-124-3p | 0.1897 (0.1430) | 0.1648 (0.2045) | 0.1790 (0.2962) | -0.2056 (0.2290) |
| miR-127-3p | 0.0915 (0.4833) | -0.0524 (0.6886) | 0.0015 (0.9932) | -0.0081 (0.9625) |
| miR-128-3p | 0.1355 (0.2978) | 0.0105 (0.9359) | 0.1170 (0.4966) | -0.0957 (0.5788) |
| miR-132-3p | 0.0969 (0.4573) | 0.1256 (0.3349) | 0.2710 (0.1099) | -0.0981 (0.5692) |
| miR-136-3p | 0.1643 (0.2057) | 0.0592 (0.6502) | -0.1143 (0.5067) | -0.1222 (0.4776) |
| miR-153-3p | -0.0762 (0.5596) | -0.1644 (0.2055) | -0.2914 (0.0846) | 0.2479 (0.1449) |
| miR-154-5p | 0.1517 (0.2433) | -0.0332 (0.7996) | -0.0578 (0.7377) | -0.0429 (0.8039) |
| miR-219a-5p | 0.0892 (0.4943) | 0.0219 (0.8670) | -0.2335 (0.1705) | 0.0283 (0.8697) |
| miR-323a-3p | 0.0479 (0.7140) | -0.0448 (0.7319) | -0.1780 (0.2991) | 0.1352 (0.4318) |
| miR-329-5p | 0.1414 (0.2769) | 0.0250 (0.8484) | -0.2216 (0.1940) | 0.1159 (0.5008) |
| miR-330-3p | -0.1075 (0.4095) | -0.1998 (0.1227) | -0.2434 (0.1525) | 0.2698 (0.1115) |
| miR-338-3p | 0.0863 (0.5084) | -0.1839 (0.1560) | -0.1651 (0.3360) | 0.1878 (0.2727) |
| miR-382-5p | 0.0242 (0.8529) | -0.0867 (0.5062) | -0.1994 (0.2437) | 0.1472 (0.3915) |
| miR-409-3p | 0.0618 (0.6360) | 0.0118 (0.9280) | -0.1896 (0.2680) | 0.1663 (0.3324) |
| miR-410-3p | **0.4052 (0.0012)** | 0.1442 (0.2677) | 0.0963 (0.5763) | -0.1794 (0.2951) |
| miR-411-5p | 0.0661 (0.6126) | 0.0493 (0.7058) | -0.1885 (0.2709) | 0.0139 (0.9358) |
| miR-432-5p | 0.0549 (0.6743) | 0.0162 (0.9011) | -0.2194 (0.1987) | 0.0339 (0.8445) |
| miR-487b-3p | 0.0714 (0.5843) | 0.0064 (0.9611) | -0.3162 (0.0603) | 0.1459 (0.3958) |
| miR-495-3p | 0.1050 (0.4206) | -0.0117 (0.9285) | -0.0360 (0.8349) | -0.1595 (0.3529) |
| miR-598-3p | -0.2392 (0.0634) | -0.0032 (0.9806) | 0.0564 (0.7438) | 0.0879 (0.6104) |
| miR-654-3p | 0.1513 (0.2446) | 0.0814 (0.5327) | -0.1688 (0.3250) | -0.0164 (0.9242) |
| miR-885-5p | -0.0222 (0.8654) | -0.0476 (0.7155) | 0.1562 (0.3629) | -0.0177 (0.9186) |
| miR-3200-3p | 0.0333 (0.7989) | 0.0546 (0.6760) | 0.0679 (0.6941) | -0.1785 (0.2977) |

|  | **subSBP** | **subDBP** | **Ratio 30:15** | **Valsalva ratio** |
| --- | --- | --- | --- | --- |
|  | Rho (p-value) | Rho (p-value) | Rho (p-value) | Rho (p-value) |
| miR-7-5p | -0.0928 (0.7325) | -0.0118 (0.9655) | -0.0140 (0.9588) | -0.1155 (0.7070) |
| miR-19b-3p | -0.1294 (0.6329) | -0.1206 (0.6564) | 0.0930 (0.7320) | 0.0633 (0.8373) |
| miR-22-3p | 0.0015 (0.9957) | 0.0029 (0.9914) | 0.1529 (0.5719) | 0.1281 (0.6766) |
| miR-29a-3p | -0.3735 (0.1541) | -0.2971 (0.2639) | 0.3528 (0.1802) | 0.1926 (0.5285) |
| miR-106a-5p | -0.1176 (0.6643) | -0.1324 (0.6251) | 0.2554 (0.3398) | 0.2421 (0.4255) |
| miR-124-3p | -0.0986 (0.7164) | -0.0515 (0.8497) | 0.4919 (0.0530) | 0.3274 (0.2749) |
| miR-127-3p | -0.2294 (0.3927) | -0.1500 (0.5792) | -0.2347 (0.3816) | -0.2008 (0.5106) |
| miR-128-3p | 0.0206 (0.9397) | 0.0529 (0.8456) | 0.1417 (0.6006) | 0.0358 (0.9077) |
| miR-132-3p | -0.4029 (0.1217) | -0.1765 (0.5133) | 0.4399 (0.0882) | 0.0000 (>0.999) |
| miR-136-3p | 0.1265 (0.6407) | -0.0118 (0.9655) | -0.1948 (0.4696) | 0.1706 (0.5774) |
| miR-153-3p | 0.1118 (0.6803) | 0.0088 (0.9741) | 0.0251 (0.9265) | 0.3549 (0.2341) |
| miR-154-5p | 0.3412 (0.1959) | 0.2706 (0.3108) | -0.4487 (0.0813) | -0.1155 (0.7070) |
| miR-219a-5p | -0.1104 (0.6841) | -0.2311 (0.3893) | 0.2171 (0.4192) | 0.2283 (0.4531) |
| miR-323a-3p | 0.0824 (0.7616) | -0.0927 (0.7327) | 0.1625 (0.5477) | 0.3003 (0.3188) |
| miR-329-5p | -0.0088 (0.9741) | -0.1118 (0.6803) | -0.0487 (0.8578) | 0.3769 (0.2043) |
| miR-330-3p | -0.3358 (0.2035) | -0.3004 (0.2582) | 0.1094 (0.6868) | 0.0688 (0.8233) |
| miR-338-3p | -0.0471 (0.8626) | -0.0618 (0.8202) | 0.4974 (0.0499) | 0.4017 (0.1737) |
| miR-382-5p | -0.1265 (0.6407) | -0.4088 (0.1159) | 0.2435 (0.3634) | 0.5475 (0.0528) |
| miR-409-3p | 0.1898 (0.4813) | -0.0751 (0.7824) | 0.2127 (0.4290) | 0.5041 (0.0790) |
| miR-410-3p | -0.4441 (0.0848) | -0.4000 (0.1248) | 0.3144 (0.2356) | 0.2889 (0.3385) |
| miR-411-5p | 0.1399 (0.6053) | -0.0412 (0.8795) | -0.1316 (0.6272) | 0.1393 (0.6499) |
| miR-432-5p | 0.0868 (0.7492) | 0.1001 (0.7123) | -0.3671 (0.1620) | -0.1846 (0.5461) |
| miR-487b-3p | 0.0559 (0.8371) | -0.1059 (0.6963) | -0.2111 (0.4326) | 0.0358 (0.9077) |
| miR-495-3p | 0.3061 (0.2489) | 0.1266 (0.6404) | -0.5820 (0.0180) | -0.2893 (0.3378) |
| miR-598-3p | 0.0721 (0.7907) | -0.0471 (0.8625) | 0.1787 (0.5078) | 0.0634 (0.8371) |
| miR-654-3p | 0.2428 (0.3648) | -0.0015 (0.9957) | -0.1396 (0.6061) | 0.1749 (0.5676) |
| miR-885-5p | -0.3814 (0.1449) | -0.3697 (0.1588) | 0.4494 (0.0808) | 0.2662 (0.3793) |
| miR-3200-3p | -0.2265 (0.3990) | -0.2441 (0.3622) | 0.2982 (0.2620) | 0.3934 (0.1836) |

|  | **SE** | **H&Y** | **GDS** | **Animal Fluency** |
| --- | --- | --- | --- | --- |
|  | Rho (p-value) | Rho (p-value) | Rho (p-value) | Rho (p-value) |
| miR-7-5p | -0.2186 (0.0905) | 0.2584 (0.0443) | 0.2394 (0.0632) | -0.0461 (0.7245) |
| miR-19b-3p | -0.1440 (0.2682) | 0.1595 (0.2195) | 0.1958 (0.1305) | 0.0243 (0.8527) |
| miR-22-3p | -0.0155 (0.9054) | 0.0542 (0.6783) | 0.0963 (0.4604) | 0.0317 (0.8086) |
| miR-29a-3p | 0.0962 (0.4606) | 0.0172 (0.8956) | -0.0602 (0.6447) | 0.0603 (0.6442) |
| miR-106a-5p | -0.1643 (0.2058) | 0.1867 (0.1496) | 0.1589 (0.2212) | 0.0413 (0.7518) |
| miR-124-3p | -0.0074 (0.9547) | 0.1228 (0.3457) | 0.0873 (0.5037) | 0.0281(0.8300) |
| miR-127-3p | 0.0131 (0.9203) | -0.0793 (0.5434) | 0.0918 (0.4817) | -0.0159 (0.9032) |
| miR-128-3p | 0.2165 (0.0938) | -0.2279 (0.0773) | 0.0004 (0.9975) | 0.2493 (0.0527) |
| miR-132-3p | 0.0288 (0.8257) | 0.0188 (0.8859) | 0.0876 (0.5022) | 0.0484 (0.7110) |
| miR-136-3p | 0.2398 (0.0627) | -0.2664 (0.0379) | -0.1813 (0.1621) | 0.1843 (0.1550) |
| miR-153-3p | 0.0199 (0.8790) | -0.0761 (0.5598) | -0.1040 (0.4250) | 0.1027 (0.4307) |
| miR-154-5p | 0.2275 (0.0778) | -0.3024 (0.0178) | -0.1543 (0.2351) | 0.1670 (0.1983) |
| miR-219a-5p | 0.1079 (0.4079) | -0.1271 (0.3291) | -0.1579 (0.2243) | 0.1403 (0.2807) |
| miR-323a-3p | 0.1729 (0.1828) | -0.2853 (0.0258) | -0.1740 (0.1798) | 0.0966 (0.4588) |
| miR-329-5p | 0.1132 (0.3850) | -0.2283 (0.0768) | -0.1645 (0.2052) | 0.0734 (0.5739) |
| miR-330-3p | 0.0972 (0.4560) | -0.1973 (0.1275) | -0.1842 (0.1554) | -0.0398 (0.7609) |
| miR-338-3p | 0.1158 (0.3740) | -0.2481 (0.0538) | -0.1558 (0.2307) | 0.0778 (0.5514) |
| miR-382-5p | 0.1804 (0.1642) | -0.2941 (0.0214) | -0.2428 (0.0593) | 0.1670 (0.1984) |
| miR-409-3p | 0.1765 (0.1736) | -0.2802 (0.0287) | -0.2071 (0.1093) | 0.0967 (0.4583) |
| miR-410-3p | 0.1577 (0.2247) | -0.2341 (0.0694) | -0.2669 (0.0376) | 0.2447 (0.0574) |
| miR-411-5p | 0.0700 (0.5919) | -0.1373 (0.2913) | -0.0167 (0.8982) | 0.1157 (0.3747) |
| miR-432-5p | 0.0401 (0.7591) | -0.1570 (0.2268) | -0.0802 (0.5388) | 0.0233 (0.8586) |
| miR-487b-3p | 0.1506 (0.2465) | -0.2595 (0.0435) | -0.1994 (0.1234) | 0.1142 (0.3806) |
| miR-495-3p | 0.2868 (0.0251) | -0.3446 (0.0065) | -0.0935 (0.4736) | 0.1846 (0.1545) |
| miR-598-3p | 0.0116 (0.9294) | 0.0589 (0.6518) | 0.1968 (0.1285) | -0.0980 (0.4525) |
| miR-654-3p | 0.0343 (0.7930) | -0.1605 (0.2166) | 0.0154 (0.9064) | 0.1575 (0.2256) |
| miR-885-5p | -0.0583 (0.6556) | 0.1184 (0.3633) | 0.0591 (0.6512) | -0.0752 (0.5645) |
| miR-3200-3p | -0.2013 (0.1198) | 0.2283 (0.0768) | 0.2850 (0.0260) | -0.1093 (0.4016) |

|  | **Digit Span Forward** | **Digit Span Backward** | **Digit Span Total** | **DBP difference in hand grip** | **DBP change in HR** |
| --- | --- | --- | --- | --- | --- |
|  | Rho (p-value) | Rho (p-value) | Rho (p-value) | Rho (p-value) | Rho (p-value) |
| miR-7-5p | 0.2893 (0.0870) | 0.2554 (0.1328) | 0.3010 (0.0744) | -0.0705 (0.8108) | 0.3384 (0.2581) |
| miR-19b-3p | 0.3001 (0.0754) | 0.2743 (0.1055) | 0.3159 (0.0605) | -0.0593 (0.8403) | 0.4154 (0.1581) |
| miR-22-3p | 0.0797 (0.6442) | 0.0656 (0.7041) | 0.0981 (0.5693) | -0.2706 (0.3494) | 0.1873 (0.5400) |
| miR-29a-3p | 0.3845 (0.0206) | 0.3196 (0.0574) | 0.3836 (0.0209) | -0.0330 (0.9109) | 0.2751 (0.3630) |
| miR-106a-5p | 0.1773 (0.3010) | 0.1377 (0.4233) | 0.1859 (0.2778) | -0.1297 (0.6586) | 0.6162 (0.0249) |
| miR-124-3p | 0.3679 (0.0273) | 0.3180 (0.0588) | 0.3738 (0.0247) | 0.0154 (0.9583) | 0.1953 (0.5225) |
| miR-127-3p | -0.0256 (0.8824) | -0.0048 (0.9778) | -0.0062 (0.9712) | 0.0769 (0.7938) | 0.3549 (0.2341) |
| miR-128-3p | 0.1198 (0.4865) | -0.0113 (0.9478) | 0.0774 (0.6537) | -0.3099 (0.2809) | 0.2228 (0.4643) |
| miR-132-3p | 0.1671 (0.3301) | 0.0787 (0.6482) | 0.1394 (0.4175) | -0.2220 (0.4456) | 0.1953 (0.5225) |
| miR-136-3p | -0.1000 (0.5618) | -0.1292 (0.4525) | -0.1203 (0.4846) | 0.0637 (0.8286) | -0.0495 (0.8724) |
| miR-153-3p | -0.3093 (0.0664) | -0.3596 (0.0312) | -0.3423 (0.0410) | 0.1473 (0.6154) | 0.3521 (0.2380) |
| miR-154-5p | -0.0571 (0.7408) | -0.0827 (0.6316) | -0.0715 (0.6784) | 0.0198 (0.9465) | -0.0880 (0.7749) |
| miR-219a-5p | -0.1144 (0.5066) | -0.1318 (0.4436) | -0.1196 (0.4873) | -0.0418 (0.8872) | 0.0303 (0.9218) |
| miR-323a-3p | -0.2511 (0.1397) | -0.2005 (0.2411) | -0.2461 (0.1479) | -0.1188 (0.6858) | 0.0275 (0.9288) |
| miR-329-5p | -0.2020 (0.2373) | -0.2400 (0.1585) | -0.2379 (0.1624) | -0.0593 (0.8403) | 0.1843 (0.5466) |
| miR-330-3p | -0.3013 (0.0741) | -0.3059 (0.0696) | -0.3159 (0.0605) | -0.0132 (0.9642) | -0.0028 (0.9929) |
| miR-338-3p | -0.1133 (0.5104) | -0.1922 (0.2615) | -0.1505 (0.3810) | -0.2264 (0.4364) | 0.1238 (0.6870) |
| miR-382-5p | -0.2623 (0.1223) | -0.2436 (0.1523) | -0.2768 (0.1022) | -0.2088 (0.4738) | 0.1100 (0.7204) |
| miR-409-3p | -0.2653 (0.1179) | -0.2038 (0.2331) | -0.2627 (0.1216) | -0.1165 (0.6917) | 0.1570 (0.6084) |
| miR-410-3p | 0.3836 (0.0209) | 0.2484 (0.1441) | 0.3423 (0.0410) | -0.4242 (0.1306) | 0.4182 (0.1550) |
| miR-411-5p | -0.1162 (0.4998) | -0.0889 (0.6061) | -0.1127 (0.5129) | 0.0616 (0.8343) | -0.3021 (0.3158) |
| miR-432-5p | -0.1493 (0.3847) | -0.1419 (0.4089) | -0.1634 (0.3411) | -0.0352 (0.9049) | -0.1488 (0.6277) |
| miR-487b-3p | -0.2651 (0.1181) | -0.2281 (0.1809) | -0.2588 (0.1274) | -0.4066 (0.1491) | 0.0385 (0.9006) |
| miR-495-3p | -0.0894 (0.6040) | -0.0714 (0.6790) | -0.0929 (0.5901) | 0.0462 (0.8755) | -0.4986 (0.0828) |
| miR-598-3p | -0.1239 (0.4714) | -0.1391 (0.4183) | -0.1253 (0.4666) | -0.4022 (0.1540) | -0.0578 (0.8513) |
| miR-654-3p | 0.0280 (0.8714) | -0.0078 (0.9639) | 0.0063 (0.9709) | -0.2552 (0.3785) | -0.0716 (0.8161) |
| miR-885-5p | 0.2934 (0.0824) | 0.2349 (0.1679) | 0.2919 (0.0841) | 0.2048 (0.4824) | 0.0869 (0.7777) |
| miR-3200-3p | 0.2122 (0.2141) | 0.1698 (0.3221) | 0.2185 (0.2004) | 0.0505 (0.8637) | 0.4072 (0.1673) |

|  | **UMSARS1** | **UMSARS2** | **UMSARS4** | **UMSARS Total** |
| --- | --- | --- | --- | --- |
|  | Rho (p-value) | Rho (p-value) | Rho (p-value) | Rho (p-value) |
| miR-7-5p | 0.0034 (0.9842) | -0.1247 (0.4687) | 0.0352 (0.8385) | -0.0749 (0.6640) |
| miR-19b-3p | -0.0736 (0.6698) | -0.1322 (0.4423) | -0.0670 (0.6980) | -0.1178 (0.4938) |
| miR-22-3p | 0.1498 (0.3832) | 0.0959 (0.5778) | 0.0764 (0.6580) | 0.1292 (0.4526) |
| miR-29a-3p | -0.0371 (0.8299) | -0.1792 (0.2956) | -0.1850 (0.2799) | -0.1163 (0.4995) |
| miR-106a-5p | -0.0245 (0.8873) | -0.0698 (0.6860) | -0.0150 (0.9310) | -0.0624 (0.7175) |
| miR-124-3p | -0.1727 (0.3139) | -0.3111 (0.0648) | -0.1874 (0.2738) | -0.2645 (0.1190) |
| miR-127-3p | -0.1023 (0.5527) | 0.0034 (0.9845) | -0.0040 (0.9816) | -0.0157 (0.9275) |
| miR-128-3p | -0.1228 (0.4756) | -0.1048 (0.5429) | -0.1148 (0.5049) | -0.1240 (0.4712) |
| miR-132-3p | 0.0037 (0.9830) | -0.0994 (0.5640) | -0.0687 (0.6903) | -0.0647 (0.7077) |
| miR-136-3p | -0.1965 (0.2507) | -0.1077 (0.5320) | -0.0476 (0.7829) | -0.1635 (0.3406) |
| miR-153-3p | 0.1333 (0.4383) | 0.2224 (0.1924) | 0.2140 (0.2102) | 0.1942 (0.2565) |
| miR-154-5p | -0.0743 (0.6665) | 0.0926 (0.5913) | 0.0628 (0.7159) | 0.0091 (0.9578) |
| miR-219a-5p | -0.1256 (0.4655) | -0.0478 (0.7817) | 0.0111 (0.9487) | -0.0855 (0.6199) |
| miR-323a-3p | -0.0774 (0.6535) | 0.1683 (0.3266) | 0.1452 (0.3982) | 0.0278 (0.8721) |
| miR-329-5p | 0.0049 (0.9774) | 0.1765 (0.3031) | 0.1346 (0.4340) | 0.0839 (0.6267) |
| miR-330-3p | -0.0415 (0.8101) | 0.2006 (0.2408) | -0.0007 (0.9967) | 0.0672 (0.6971) |
| miR-338-3p | -0.0829 (0.6307) | 0.2414 (0.1560) | 0.1071 (0.5340) | 0.0431 (0.8027) |
| miR-382-5p | -0.0708 (0.6816) | 0.1942 (0.2564) | 0.1385 (0.4206) | 0.0375 (0.8282) |
| miR-409-3p | 0.0006 (0.9973) | 0.1858 (0.2780) | 0.1297 (0.4510) | 0.0740 (0.6680) |
| miR-410-3p | -0.2719 (0.1087) | -0.2059 (0.2283) | -0.1648 (0.3367) | -0.2677 (0.1145) |
| miR-411-5p | -0.0036 (0.9833) | 0.1085 (0.5289) | 0.1556 (0.3647) | 0.0633 (0.7139) |
| miR-432-5p | -0.0294 (0.8647) | 0.0990 (0.5656) | 0.1164 (0.4990) | 0.0297 (0.8636) |
| miR-487b-3p | -0.0460 (0.7899) | 0.1968 (0.2501) | 0.1778 (0.2996) | 0.0693 (0.6881) |
| miR-495-3p | -0.2111 (0.2166) | -0.1090 (0.5270) | -0.0754 (0.6622) | -0.1477 (0.3900) |
| miR-598-3p | 0.1050 (0.5421) | 0.1329 (0.4397) | 0.1505 (0.3810) | 0.1345 (0.4343) |
| miR-654-3p | 0.0178 (0.9180) | 0.1266 (0.4619) | 0.1142 (0.5074) | 0.0511 (0.7671) |
| miR-885-5p | 0.1363 (0.4280) | -0.0840 (0.6261) | -0.0595 (0.7303) | 0.0279 (0.8717) |
| miR-3200-3p | -0.1115 (0.5175) | -0.1340 (0.4357) | -0.0114 (0.9474) | -0.1353 (0.4313) |

|  | **SCOPA Gastro** | **SCOPA Urinary** | **SCOPA Cardio** | **SCOPA Thermo** | **SCOPA Pupillo** | **SCOPA Sexual Men** | **SCOPA Total** |
| --- | --- | --- | --- | --- | --- | --- | --- |
|  | Rho (p-value) | Rho (p-value) | Rho (p-value) | Rho (p-value) | Rho (p-value) | Rho (p-value | Rho (p-value) |
| miR-7-5p | 0.1778 (0.1780) | 0.2159 (0.1005) | 0.1837 (0.1637) | -0.0408 (0.7589) | 0.0924 (0.4865) | 0.1002 (0.5918) | 0.2021 (0.1248) |
| miR-19b-3p | 0.1781 (0.1772) | 0.1555 (0.2396) | 0.1006 (0.4486) | -0.0004 (0.9974) | 0.1830 (0.1653) | 0.1078 (0.5639) | 0.1458 (0.2706) |
| miR-22-3p | 0.0080 (0.9521) | -0.0019 (0.9888) | 0.1160 (0.3816) | -0.0922 (0.4872) | -0.0167 (0.9002) | -0.0817 (0.6620) | -0.0587 (0.6587) |
| miR-29a-3p | 0.0162 (0.9029) | -0.0134 (0.9199) | -0.0225 (0.8656) | -0.1748 (0.1853) | -0.1826 (0.1662) | 0.2871 (0.1173) | -0.0355 (0.7896) |
| miR-106a-5p | 0.1745 (0.1862) | 0.1055 (0.4263) | 0.0146 (0.9127) | 0.0009 (0.9943) | 0.1981 (0.1325) | 0.0812 (0.6641) | 0.1223 (0.3563) |
| miR-124-3p | 0.1793 (0.1742) | 0.2029 (0.1232) | 0.2535 (0.0527) | -0.0986 (0.4573) | -0.0115 (0.9310) | 0.2576 (0.1618) | 0.1596 (0.2271) |
| miR-127-3p | -0.0663 (0.6177) | -0.3009 (0.0206) | -0.2931 (0.0243) | -0.0511 (0.7009) | -0.0544 (0.6823) | -0.3223 (0.0770) | -0.1752 (0.1844) |
| miR-128-3p | -0.0038 (0.9773) | -0.1240 (0.3494) | -0.1570 (0.2349) | -0.0713 (0.5913) | -0.1621 (0.2200) | -0.1591 (0.3925) | -0.1212 (0.3605) |
| miR-132-3p | 0.2105 (0.1096) | 0.1219 (0.3579) | 0.1933 (0.1423) | 0.0960 (0.4696) | 0.1585 (0.2305) | 0.2976 (0.1040) | 0.1854 (0.1597) |
| miR-136-3p | -0.2560 (0.0504) | -0.3437 (0.0077) | -0.3571 (0.0055) | -0.0999 (0.4515) | -0.1303 (0.3253) | -0.5065 (0.0036) | -0.3908 (0.0022) |
| miR-153-3p | -0.1399 (0.2908) | -0.3548 (0.0058) | -0.3010 (0.0205) | -0.0244 (0.8545) | -0.0162 (0.9033) | -0.3258 (0.0737) | -0.3470 (0.0071) |
| miR-154-5p | -0.3539 (0.0060) | **-0.4327 (0.0006)** | -0.2341 (0.0744) | -0.0438 (0.7421) | -0.0555 (0.6766) | -0.4589 (0.0094) | **-0.4817 (0.0001)** |
| miR-219a-5p | -0.1633 (0.2164) | -0.1999 (0.1290) | -0.2786 (0.0326) | -0.2566 (0.0498) | -0.2880 (0.0269) | -0.3357 (0.0648) | -0.2985 (0.0216) |
| miR-323a-3p | -0.3185 (0.0140) | **-0.4952 (0.0001)** | -0.2822 (0.0304) | 0.1876 (0.1548) | 0.1126 (0.3957) | **-0.5809 (0.0006)** | **-0.4548 (0.0003)** |
| miR-329-5p | -0.1845 (0.1619) | -0.3299 (0.0107) | -0.1272 (0.3371) | 0.1584 (0.2308) | 0.0862 (0.5165) | -0.3884 (0.0308) | -0.2907 (0.0255) |
| miR-330-3p | -0.2076 (0.1146) | -0.2470 (0.0593) | -0.3303 (0.0106) | -0.0411 (0.7572) | -0.2133 (0.1048) | -0.2123 (0.2514) | -0.3024 (0.0199) |
| miR-338-3p | -0.2485 (0.0577) | -0.3876 (0.0024) | -0.2896 (0.0261) | 0.0137 (0.9177) | -0.0380 (0.7748) | -0.3269 (0.0727) | **-0.4169 (0.0010)** |
| miR-382-5p | -0.3339 (0.0098) | -0.3892 (0.0023) | -0.2634 (0.0438) | 0.0779 (0.5575) | 0.0205 (0.8776) | -0.3995 (0.0260) | **-0.4465 (0.0004)** |
| miR-409-3p | -0.2746 (0.0353) | **-0.4418 (0.0005)** | -0.2619 (0.0451) | 0.1461 (0.2695) | 0.0405 (0.7607) | -0.4814 (0.0061) | -0.3898 (0.0023) |
| miR-410-3p | -0.1311 (0.3225) | -0.3136 (0.0156) | -0.0045 (0.9727) | -0.0543 (0.6827) | -0.0500 (0.7067) | -0.2957 (0.1062) | -0.3229 (0.0126) |
| miR-411-5p | -0.1177 (0.3747) | -0.3021 (0.0200) | -0.2761 (0.0343) | 0.0580 (0.6628) | 0.0616 (0.6429) | **-0.5442 (0.0016)** | -0.2658 (0.0419) |
| miR-432-5p | -0.1481 (0.2631) | -0.3038 (0.0193) | -0.1915 (0.1463) | 0.1114 (0.4009) | 0.1054 (0.4268) | -0.5260 (0.0024) | -0.2253 (0.0862) |
| miR-487b-3p | -0.3197 (0.0136) | **-0.4546 (0.0003)** | -0.3427 (0.0079) | 0.0170 (0.8986) | -0.0570 (0.6680) | **-0.6165 (0.0002)** | **-0.4229 (0.0008)** |
| miR-495-3p | -0.2301 (0.0795) | **-0.4354 (0.0006)** | -0.3365 (0.0092) | -0.0518 (0.6969) | -0.0748 (0.5735) | -0.4870 (0.0055) | -0.3916 (0.0022) |
| miR-598-3p | 0.2579 (0.0486) | 0.1013 (0.4450) | -0.0126 (0.9246) | -0.0130 (0.9219) | 0.0956 (0.4713) | 0.0608 (0.7454) | 0.1964 (0.1360) |
| miR-654-3p | 0.0374 (0.7786) | -0.1182 (0.3724) | 0.0063 (0.9625) | 0.1776 (0.1783) | 0.1992 (0.1304) | -0.0695 (0.7104) | -0.0916 (0.4903) |
| miR-885-5p | 0.1390 (0.2936) | 0.0973 (0.4634) | 0.1356 (0.3060) | -0.0858 (0.5183) | -0.1030 (0.4375) | 0.2441 (0.1857) | 0.1013 (0.4452) |
| miR-3200-3p | 0.1912 (0.1468) | 0.2187 (0.0961) | 0.1815 (0.1688) | -0.0984 (0.4583) | 0.0485 (0.7154) | 0.2097 (0.2576) | 0.1905 (0.1484) |

|  | **GB Imitation**  **Total80** | **GB Hand Imitation MAX40** | **GB Finger Imitation MAX40** | **GB Pantomime Score MAX55** | **GB Grip MAX20** | **GB Position Movement MAX35** |
| --- | --- | --- | --- | --- | --- | --- |
|  | Rho (p-value) | Rho (p-value) | Rho (p-value) | Rho (p-value) | Rho (p-value) | Rho (p-value) |
| miR-7-5p | 0.1851 (0.2798) | 0.2259 (0.1852) | 0.1186 (0.4909) | 0.2316 (0.1742) | 0.0775 (0.6533) | 0.2310 (0.1753) |
| miR-19b-3p | 0.1661 (0.3329) | 0.1761 (0.3044) | 0.1154 (0.5028) | 0.2065 (0.2270) | 0.1011 (0.5574) | 0.2081 (0.2233) |
| miR-22-3p | 0.0634 (0.7133) | 0.0921 (0.5933) | 0.0082 (0.9619) | 0.1572 (0.3597) | -0.0426 (0.8050) | 0.1996 (0.2433) |
| miR-29a-3p | 0.2708 (0.1101) | 0.2308 (0.1757) | 0.2394 (0.1597) | 0.0932 (0.5888) | -0.0151 (0.9306) | 0.0849 (0.6225) |
| miR-106a-5p | 0.1182 (0.4923) | 0.1067 (0.5357) | 0.0955 (0.5796) | 0.1523 (0.3751) | 0.0409 (0.8129) | 0.1675 (0.3289) |
| miR-124-3p | 0.2129 (0.2124) | 0.2663 (0.1164) | 0.1252 (0.4668) | 0.2295 (0.1782) | 0.0635 (0.7130) | 0.2398 (0.1590) |
| miR-127-3p | 0.0921 (0.5933) | 0.0483 (0.7798) | 0.1085 (0.5287) | -0.2093 (0.2205) | -0.0395 (0.8190) | -0.2653 (0.1179) |
| miR-128-3p | 0.0613 (0.7224) | 0.0284 (0.8696) | 0.0676 (0.6951) | 0.0385 (0.8234) | -0.0823 (0.6333) | 0.1416 (0.4099) |
| miR-132-3p | 0.1899 (0.2672) | 0.1223 (0.4772) | 0.2164 (0.2049) | 0.3456 (0.0390) | 0.0488 (0.7774) | 0.4438 (0.0067) |
| miR-136-3p | -0.0078 (0.9639) | -0.0626 (0.7166) | 0.0856 (0.6198) | -0.1961 (0.2516) | 0.0953 (0.5805) | -0.2733 (0.1067) |
| miR-153-3p | -0.2751 (0.1044) | -0.2474 (0.1458) | -0.2515 (0.1390) | -0.3468 (0.0382) | -0.1139 (0.5083) | -0.3910 (0.0184) |
| miR-154-5p | -0.0102 (0.9531) | -0.0811 (0.6382) | 0.0517 (0.7647) | -0.1434 (0.4040) | 0.0121 (0.9443) | -0.1835 (0.2840) |
| miR-219a-5p | -0.0746 (0.6653) | -0.0441 (0.7983) | -0.1308 (0.4471) | -0.3812 (0.0218) | -0.0973 (0.5724) | -0.4426 (0.0069) |
| miR-323a-3p | -0.1412 (0.4114) | -0.1912 (0.2639) | -0.0487 (0.7781) | -0.1984 (0.2462) | -0.0032 (0.9850) | -0.2443 (0.1510) |
| miR-329-5p | -0.2299 (0.1773) | -0.2737 (0.1062) | -0.1400 (0.4155) | -0.0794 (0.6453) | 0.0689 (0.6899) | -0.1029 (0.5505) |
| miR-330-3p | -0.2677 (0.1144) | -0.3669 (0.0277) | -0.1245 (0.4693) | -0.1722 (0.3153) | -0.0732 (0.6712) | -0.1694 (0.3232) |
| miR-338-3p | -0.2182 (0.2010) | -0.2690 (0.1127) | -0.1908 (0.2650) | -0.2454 (0.1491) | -0.1666 (0.3315) | -0.2154 (0.2070) |
| miR-382-5p | -0.2648 (0.1186) | -0.2700 (0.1112) | -0.1663 (0.3322) | -0.0897 (0.6029) | 0.0335 (0.8463) | -0.1002 (0.5611) |
| miR-409-3p | -0.1449 (0.3992) | -0.1958 (0.2524) | -0.0467 (0.7868) | -0.2109 (0.2170) | -0.0918 (0.5942) | -0.2167 (0.2043) |
| miR-410-3p | 0.1389 (0.4191) | 0.1265 (0.4623) | 0.1228 (0.4754) | 0.1745 (0.3087) | 0.1631 (0.3417) | 0.1341 (0.4354) |
| miR-411-5p | -0.0910 (0.5975) | -0.1348 (0.4332) | -0.0436 (0.8007) | -0.1883 (0.2715) | -0.0546 (0.7517) | -0.1949 (0.2547) |
| miR-432-5p | -0.1523 (0.3753) | -0.1794 (0.2950) | -0.0816 (0.6363) | -0.1056 (0.5401) | -0.0151 (0.9305) | -0.0938 (0.5862) |
| miR-487b-3p | -0.2349 (0.1679) | -0.2674 (0.1148) | -0.1366 (0.4271) | -0.3386 (0.0434) | -0.1018 (0.5548) | -0.3903 (0.0186) |
| miR-495-3p | 0.0846 (0.6236) | 0.0795 (0.6448) | 0.1214 (0.4806) | -0.2083 (0.2227) | -0.0378 (0.8266) | -0.2258 (0.1855) |
| miR-598-3p | -0.0513 (0.7666) | -0.0451 (0.7942) | -0.0737 (0.6691) | 0.0616 (0.7214) | -0.1015 (0.5557) | 0.1527 (0.3740) |
| miR-654-3p | -0.1233 (0.4736) | -0.1903 (0.2662) | -0.0849 (0.6226) | -0.1415 (0.4104) | -0.1461 (0.3953) | -0.0855 (0.6202) |
| miR-885-5p | 0.1501 (0.3822) | 0.1644 (0.3381) | 0.1250 (0.4675) | 0.1980 (0.2470) | 0.0847 (0.6233) | 0.1613 (0.3472) |
| miR-3200-3p | 0.1036 (0.5477) | 0.1812 (0.2902) | 0.0083 (0.9616) | 0.0616 (0.7211) | -0.0197 (0.9094) | 0.0575 (0.7390) |

**Supplementary Table 5. Dysregulated KEGG pathways associated with miRNA target genes.** The union of all dysregulated miRNA target genes was analyzed using the DIANA-miRPath v3.0 web server with default parameters (microT-CDS threshold: 0.8, p-value threshold: 0.05, FDR correction applied). The number of genes regulated by a specific miRNA for each pathway is indicated in parentheses. Note that multiple miRNAs may regulate the same mRNA target.

| **KEGG pathway** | ***p* value** | ***#* genes** | **miRNAs** |
| --- | --- | --- | --- |
| Prion diseases | 2.7×10^-11^ | 11 | miR-409-3p (1), miR-495-3p (9), miR-330-3p (2), miR-153-3p (2) |
| Hippo signaling pathway | 8.4×10^-7^ | 60 | miR-329-5p (2), miR-330-3p (13), miR-495-3p (46), miR-409-3p (9), miR-411-5p (3), miR-136-3p (2), miR-219a-5p (1), miR-323a-3p (6), miR-153-3p (6), miR-3200-3p (1) |
| Mucin-type O-Glycan biosynthesis | 1.2×10^-6^ | 12 | miR-153-3p (3), miR-330-3p (1), miR-495-3p (5), miR-323a-3p (4), miR-409-3p (2) |
| Adherens junction | 2.0×10^-6^ | 34 | miR-409-3p (4), miR-495-3p (24), miR-330-3p (4), miR-323a-3p (5), miR-153-3p (7), miR-219a-5p (1) |
| Wnt signaling pathway | 2.5×10^-6^ | 61 | miR-136-3p (1), miR-330-3p (12), miR-495-3p (47), miR-219a-5p (2), miR-153-3p (12), miR-329-5p (1), miR-323a-3p (4), miR-411-5p (3), miR-409-3p (1) |
| TGF-beta signaling pathway | 3.0×10^-6^ | 36 | miR-409-3p (5), miR-495-3p (31), miR-330-3p (7), miR-153-3p (9), miR-323a-3p (4), miR-329-5p (1), miR-136-3p (1) |
| Ubiquitin mediated proteolysis | 2.5×10^-5^ | 56 | miR-409-3p (6), miR-495-3p (46), miR-153-3p (10), miR-323a-3p (7), miR-330-3p (7), miR-411-5p (2), miR-154-5p (1), miR-136-3p (1), miR-3200-3p (1) |
| FoxO signaling pathway | 3.0×10^-5^ | 57 | miR-495-3p (41), miR-154-5p (1), miR-330-3p (8), miR-153-3p (10), miR-323a-3p (10), miR-409-3p (7), miR-411-5p (1), miR-3200-3p (1), miR-329-5p (1), miR-136-3p (2) |
| Axon guidance | 3.3×10^-5^ | 51 | miR-330-3p (8), miR-495-3p (35), miR-323a-3p (9), miR-409-3p (6), miR-136-3p (1), miR-153-3p (17), miR-154-5p (1), miR-219a-5p (1), miR-487b-3p (1), miR-329-5p (1) |
| ErbB signaling pathway | 5.5×10^-5^ | 42 | miR-323a-3p (9), miR-495-3p (27), miR-330-3p (11), miR-329-5p (2), miR-409-3p (4), miR-153-3p (6), miR-136-3p (1), miR-487b-3p (1) |
| Long-term potentiation | 5.5×10^-5^ | 34 | miR-323a-3p (7), miR-409-3p (6), miR-495-3p (23), miR-329-5p (2), miR-153-3p (5), miR-411-5p (1), miR-330-3p (8), miR-136-3p (1) |
| Focal adhesion | 0.0001 | 79 | miR-495-3p (53), miR-154-5p (1), miR-330-3p (15), miR-153-3p (14), miR-323a-3p (8), miR-409-3p (7), miR-329-5p (2), miR-487b-3p (1), miR-136-3p (2), miR-3200-3p (3) |
| Oxytocin signaling pathway | 0.0002 | 61 | miR-153-3p (17), miR-495-3p (43), miR-323a-3p (11), miR-411-5p (5), miR-3200-3p (1), miR-330-3p (11), miR-409-3p (6), miR-154-5p (1), miR-329-5p (3), miR-219a-5p (1) |
| Circadian rhythm | 0.0002 | 18 | miR-323a-3p (4), miR-330-3p (6), miR-409-3p (2), miR-495-3p (16), miR-411-5p (1) |
| Gap junction | 0.0003 | 32 | miR-495-3p (20), miR-153-3p (8), miR-154-5p (1), miR-323a-3p (7), miR-329-5p (3), miR-330-3p (8), miR-411-5p (2), miR-409-3p (3) |
| Long-term depression | 0.0005 | 25 | miR-153-3p (8), miR-330-3p (7), miR-495-3p (18), miR-409-3p (5), miR-323a-3p (5), miR-136-3p (1), miR-154-5p (1), miR-329-5p (2), miR-411-5p (1) |
| Phosphatidylinositol signaling system | 0.0008 | 30 | miR-495-3p (21), miR-153-3p (7), miR-330-3p (6), miR-323a-3p (5), miR-409-3p (3), miR-3200-3p (1), miR-411-5p (1), miR-329-5p (1) |
| Sphingolipid signaling pathway | 0.0008 | 45 | miR-153-3p (12), miR-323a-3p (8), miR-495-3p (30), miR-154-5p (1), miR-409-3p (5), miR-330-3p (5), miR-329-5p (2), miR-411-5p (1), miR-136-3(1) |
| Estrogen signaling pathway | 0.0010 | 37 | miR-153-3p (8), miR-323a-3p (8), miR-330-3p (7), miR-495-3p (23), miR-409-3p (4), miR-411-5p (2), miR-3200-3p (1), miR-329-5p (3) |
| Amphetamine addiction | 0.0010 | 27 | miR-495-3p (22), miR-323a-3p (6), miR-409-3p (4), miR-153-3(3), miR-330-3p (6), miR-329-5p (1) |
| Dopaminergic synapse | 0.0010 | 52 | miR-495-3p (38), miR-329-5p (4), miR-323a-3p (10), miR-330-3p (7), miR-409-3p (8), miR-153-3p (7), miR-411-5p (1), miR-3200-3p (1), miR-136-3p (1) |
| AMPK signaling pathway | 0.0018 | 47 | miR-330-3p (5), miR-495-3p (36), miR-409-3p (8), miR-153-3p (9), miR-136-3(2), miR-323a-3p (7), miR-411-5p (2), miR-329-5p (2) |
| Prolactin signaling pathway | 0.0019 | 28 | miR-323a-3p (6), miR-409-3p (3), miR-495-3p (16), miR-153-3p (4), miR-330-3p (6), miR-329-5p (2), miR-3200-3p (1) |
| Thyroid hormone signaling pathway | 0.0020 | 46 | miR-330-3p (12), miR-3200-3p (2), miR-323a-3p (6), miR-495-3p (33), miR-219a-5p (2), miR-153-3p (7), miR-329-5p (2), miR-409-3p (2) |
| GABAergic synapse | 0.0021 | 28 | miR-411-5p (2), miR-153-3p (6), miR-136-3(2), miR-323a-3p (5), miR-495-3p (18), miR-409-3p (2), miR-330-3p (4), miR-3200-3p (1) |
| Rap1 signaling pathway | 0.0024 | 71 | miR-495-3p (47), miR-153-3p (10), miR-323a-3p (7), miR-330-3p (16), miR-409-3p (9), miR-136-3p (2), miR-411-5p (4), miR-3200-3p (1), miR-329-5p (3), miR-487b-3p (1) |
| Glutamatergic synapse | 0.00277 | 39 | miR-495-3p (28), miR-153-3p (9), miR-330-3p (5), miR-323a-3p (7), miR-409-3p (4), miR-411-5p (1), miR-329-5p (1), miR-219a-5p (1) |
